# A respiratory syncytial virus trailer sequence modulates viral replication and the generation and propagation kinetics of copy-back defective viral genomes

**DOI:** 10.1128/jvi.00184-26

**Published:** 2026-03-24

**Authors:** Justin W. Brennan, Gaochan Wang, Sarah Connor, Xingjian Wang, Thomas J. Mariani, Yan Sun

**Affiliations:** 1 Department of Microbiology & Immunology, University of Rochester Medical Center 6923, Rochester, New York, USA; 2 Division of Allergy, Immunology, and Rheumatology (AIR), Department of Medicine, University of Rochester Medical Center 6923, Rochester, New York, USA; 3 Department of Pediatrics, Center for Children’s Health Research, University of Rochester Medical Center 6923, Rochester, New York, USA; University of Kentucky College of Medicine, Lexington, Kentucky, USA

**Keywords:** defective viral genome generation and accumulation, respiratory syncytial virus infection, virus variant, virus passaging, type I/III interferon responses, trailer sequence

## Abstract

Copy-back defective viral genomes (cbDVGs) are key inducers of antiviral responses during negative-sense RNA virus infection. Once considered byproducts of *in vitro* viral replication, cbDVGs have since been detected in clinical specimens and implicated in affecting infection outcomes. The molecular mechanism of cbDVG generation remains unclear, thereby hindering our ability to manipulate cbDVG production during infection for therapeutic gain. Previous work showed that respiratory syncytial virus (RSV) cbDVG re-initiation sites cluster in trailer-end hotspots R1, R2, and R3, and that a poly-U mutation in R1 selectively reduced cbDVG formation at the mutated region. Here, we report that a 10U mutation in R2 drastically reduced cbDVGs in this region in both minigenome and recombinant virus systems. Furthermore, during high-multiplicity of infection passaging of the R2-10U virus, we observed delayed detection of cbDVGs with re-initiation sites in R1–R3 (trailer cbDVGs) compared to WT, while no differences in virus titers were observed. Interestingly, we observed the rapid emergence and accumulation of a viral variant bearing a 2-ribonucleotide deletion (R2-8U) within the R2-10U mutation sequence as early as P0. Compared to R2-10U, the R2-8U virus was stable, displayed faster generation and accumulation of trailer cbDVGs, restored cbDVGs with R2 re-initiation sites, and exhibited enhanced genomic replication. Overall, our data identify a sequence in the RSV trailer whose mutation critically modulates both viral replication and the generation/propagation of trailer cbDVGs. Our data also suggest that cbDVG generation, particularly near the trailer, may be an evolutionary tradeoff for more rapid virus genomic replication.

**IMPORTANCE:**

Copy-back defective viral genomes (cbDVGs) are potent inducers of antiviral responses during negative-sense RNA virus infection. They have also been detected in clinical specimens and implicated in modulating infection outcomes. However, the molecular mechanisms governing cbDVG generation remain poorly understood, limiting efforts to manipulate their production for therapeutic benefit. In this manuscript, we focus on cbDVGs generated near the viral trailer end during respiratory syncytial virus (RSV) infection and identify a sequence within the RSV trailer region that, when mutated, critically alters both viral genomic replication and trailer cbDVG generation and propagation. Our observations support the notion that variables promoting enhanced levels of viral replication promote trailer cbDVG emergence and accumulation. Collectively, this work expands the repertoire of RSV genetic tools to manipulate cbDVG composition and kinetics, providing a unique platform to investigate RSV genomic replication, cbDVG-mediated pathogenesis, and the evolutionary significance of cbDVGs.

## INTRODUCTION

Defective viral genomes (DVGs) are truncated derivatives of their parental viruses, generated during an aberrant round of viral genomic replication, and are unable to replicate in the absence of a co-infecting, homologous, standard virus. DVGs have been observed during infections with many RNA viruses (1–8). They are well-documented triggers of antiviral innate immune responses, and certain DVGs interfere with the replication of their standard viruses by competing for critical viral proteins required for genomic replication or packaging into the virus particle (1, 3, 6, 9–19). Recent reports have detected DVGs in clinical samples from individuals infected with SARS-CoV-2, influenza A, dengue, measles, and respiratory syncytial virus (RSV) (3, 14, 20–24). Importantly, the amount and kinetics of DVG production are associated with infection outcomes (23, 25). For example, a non-pathogenic influenza A virus, identified from mild human infections, accumulated high levels of DVGs *in vitro* and exhibited reduced pathogenicity in mice (25). Likewise, a longitudinal cohort study of adults experimentally infected with RSV showed that individuals with late or prolonged detection of DVGs had worse symptom severity scores and higher viral loads than individuals with early DVG detection, highlighting the critical role of DVG kinetics in influencing the morbidity of viral infection (23). Despite intensive studies on the functions of DVGs, the molecular basis of DVG generation is largely unknown.

Two major classes of DVGs, deletion- and copy-back (cbDVG) DVGs, are consistently observed during viral infections. While positive-sense RNA viruses and influenza viruses produce the deletion type of DVG (3, 24, 26–29), single-stranded, negative-sense RNA viruses, like RSV, mainly produce the copy-back type of DVG (8, 21–23, 30, 31). cbDVGs are generated during an aberrant round of viral genomic replication when it is thought that the viral polymerase dissociates from the antigenomic template strand at a “break point” and reinitiates RNA synthesis at a “rejoin point” on the nascent strand and continues elongating through the 5′ end (32, 33). This leads to the generation of a theoretical panhandle RNA species containing reverse complementary termini and a unique break and rejoin junction sequence (34–36). We previously found that RSV cbDVGs with rejoin points located near and within the trailer complement sequence, spanning the last ~200 nucleotides of the antigenome, are selectively enriched during high multiplicity of infection (MOI) passaging *in vitro* (8). In virus stocks enriched with these trailer cbDVGs, cbDVGs induce robust type I and III interferon (IFN) responses, reduce viral load, and alleviate lung pathology *in vivo* (13). Notably, trailer cbDVGs have also been identified in nasal samples from hospitalized pediatric patients with high viral loads (14). To investigate the viral factors that determine trailer cbDVG generation and accumulation during infection, we divided the antigenomic region clustered with cbDVG rejoin points into three hotspots: R1, R2, and R3. R1 spans the end of the L gene, including the L gene end signal, while R2 and R3 are located within the trailer complement sequence. Previously, we mutated a short sequence containing G/C ribonucleotides to a poly-U sequence within R1 and found that this mutation reduced the abundance of cbDVGs containing rejoin points within R1, suggesting that the composition of cbDVG populations can be genetically manipulated by altering rejoin hotspot sequences (8). However, the impact of mutations in other rejoin point hotspots, the influence of rejoin hotspot mutations on cbDVG kinetics, and whether different compositions of cbDVG populations vary in their ability to trigger antiviral responses or interfere with their standard viruses remain unclear. In this study, we sought to examine these questions by specifically assessing the impact of R2 mutations on cbDVG production.

Here, we first introduced a 10U mutation into the R2 region in a recombinant RSV (R2-10U virus). Consistent with the poly-U mutation in R1, we observed a dramatic loss of cbDVGs containing R2 rejoin points during infection. Moreover, this mutation resulted in a distinct delay in the initial detection of trailer cbDVGs compared to a recombinant WT virus. Interestingly, we observed the rapid emergence and accumulation of a genotype variant bearing a 2-ribonucleotide deletion within the R2-10U mutation sequence, yielding an R2-8U virus. We then generated an R2-8U recombinant virus to examine its impact on viral replication and cbDVG dynamics. Compared to the R2-10U virus, the R2-8U genotype was stable, had enhanced replication kinetics, and correspondingly showed enhanced generation and/or propagation kinetics of cbDVGs with rejoin points within R1–R3. Functionally, we found no differences in the IFN responses induced by virus stocks enriched with cbDVGs predominately comprising R2 or R3 cbDVGs. Overall, these findings identify a critical RSV trailer sequence whose mutation modulates both viral replication and cbDVG dynamics and suggest that cbDVG generation, particularly near the trailer, may be an evolutionary tradeoff for rapid viral replication.

## MATERIALS AND METHODS

### Cells and viruses

HEp2, A549, HEK293T, and BSRT7 cells were cultured at 37°C and 5% CO_2_ with Dulbecco’s modified Eagle’s medium (Gibco) containing 10% fetal bovine serum (FBS), 1 mM sodium pyruvate (Gibco), 2 mM L-glutamate (Gibco), and 50 μg/mL gentamicin (Gibco). Baby hamster kidney cells, constitutively expressing the T7 polymerase (BSRT7 cells), were additionally cultured with 500 μg/mL geneticin (Gibco). All cells were treated with mycoplasma removal agent before experimentation (MP Biomedicals). A549 cells constitutively expressing the RSV N protein (A549-RSV-N) were generated by lentiviral transduction using the pLVX-EF1α-IRES-Puro (Takara) lentiviral expression vector, followed by puromycin selection and single clone propagation. A549-RSV-N cells were cultured in 10% FBS tissue culture media supplemented with 1.5 μg/mL puromycin. All recombinant viruses were generated using the RSV reverse genetic system in BSRT7 cells. Viral amplification was performed in HEp2 cells. Viral titration was performed in HEp2 cells or BSRT7 cells, where indicated.

### Plasmids

Mammalian expression vectors for RSV N (NR-36462), P (NR-36463), M2-1 (NR-36464), and L (NR-36461) proteins, and the RSV reverse genetic backbone pSynkRSV-line19F (NR-36460) were obtained from BEI Resources. The RSV minigenome constructs used to assess the impact of mutations on cbDVG generation have been previously described (8). R2-10U and R2-8U mutations were introduced into the pSynkRSV-line19F reverse genetic system backbone by site-directed mutagenesis. To clone specific RSV cbDVG species, the BsiWI restriction enzyme sequence present in the RSV trailer sequence was utilized. First, site-directed mutagenesis (Agilent) was used to insert the NcoI restriction enzyme site immediately following the T7 promoter in the RSV minigenome R1 construct (8). cbDVG 1563 was reverse transcribed from the total RNA of the passage 9 (P9) lineage 1 R2-10U virus using Small-Rev. cbDVG 1563 cDNA was further amplified using NcoI-HhRbz-RevSmall, targeting the end of the RSV trailer sequence with a 5′ overhang containing the NcoI restriction site and the hammerhead ribozyme sequence, and BsiWI-2-R containing the BsiWI restriction site (see Table S1). The cbDVG 1563 amplicon and the R1 minigenome constructs were restriction digested with NcoI and BsiWI, followed by ligation. Sanger sequencing of cbDVG 1563 cDNA indicated that it contained an R2-8U mutation, which was introduced into the cbDVG 1563-expressing plasmid by site-directed mutagenesis (Agilent). cbDVG 1887 was reverse transcribed from the total RNA of the 10 lineage 2 WT virus using BsiWI-R, and PCR amplification was performed using NcoI-HhRbz-RevSmall and BsiWI-2-R primers, followed by ligation into the digested vector.

### RSV minigenome system

BSRT7 cells were transfected with the RSV minigenome construct plasmids along with the codon-optimized plasmids encoding RSV: N, P, M2-1, and the L proteins, respectively. Lipofectamine 2000 and plasmids (3:1 lipofectamine 2000 to DNA ratio) were incubated at room temperature for 20 minutes, and cells were incubated with transfection complexes at room temperature for 2 hours, shaking at 250 rpm. Cells were incubated overnight at 37°C in Opti-MEM. The following morning, the cell media were replaced with antibiotic-free tissue culture media containing 2% FBS. Cells were harvested 48 hours post-transfection for RNA extraction. Extracted RNA was treated with Turbo DNase I, and RNA was re-extracted prior to cbDVG RT-PCR analysis.

### RSV reverse genetic system

BSRT7 cells were transfected with pSynkRSV-line19F (WT), R2-10U, or R2-8U along with the codon-optimized plasmids encoding RSV N, P, M2-1, and the L proteins, respectively. Lipofectamine 2000 and plasmids (3:1 lipofectamine 2000 to DNA ratio) were incubated at room temperature for 20 minutes, and cells were incubated with transfection complexes at room temperature for 2 hours, shaking at 250 rpm. Cells were incubated overnight at 37°C in Opti-MEM. The following morning, the cell media was replaced with antibiotic-free tissue culture media containing 2% FBS. Three days post-transfection, cells were split and maintained in 2% FBS tissue culture media until 50% of cells were mKate2 positive. This was repeated for two additional passages, at which time the recombinant viruses were collected. Viruses recovered from the transfection are referred to as passage 0 (P0). The total time from transfection to the collection of P0 viruses was 12 days.

### RSV serial passaging

HEp2 cells were infected with 600 µL of P0 for 5 days to generate P1. HEp2 cells were infected with P1 virus at an MOI of 0.1 for 4 days to generate P2. cbDVG enrichment began at P3. Virus lineages were generated by infecting separate sets of HEp2 cells at an MOI of 5 with P2 viruses or by rescuing identical viruses from independent instances of the RSV reverse genetic system. Subsequent passages were generated by infecting HEp2 cells at an MOI of 5 with the preceding passage for each virus lineage for three or seven additional passages. Each MOI 5 passage proceeded for 3 days. cbDVG enrichment was confirmed by RSV cbDVG RT-PCR. To generate virus stocks with low cbDVG content, P2 viruses were passaged two or three times in HEp2 cells at an MOI of 0.001 for 5 days per passage to generate the P4 and P5 low DVG content (LD) viruses, respectively. RSV cbDVG depletion was confirmed by cbDVG RT-PCR. Virus titer was calculated by determining the fluorescent-forming units (FFUs)/mL. Briefly, HEp2 cells were infected with serially diluted viral supernatants, and fluorescent cells were quantified 24 hours post-infection. Cellular fractions underwent three cycles of quick freezing and thawing prior to viral FFU/mL determination.

### Identification of cbDVGs by RNA deep sequencing and ViReMa/VODKA

RNA was extracted from designated viral stocks via Trizol-LS according to the manufacturer’s instructions. Quality control assessments of RNA samples were performed with the Agilent Bioanalyzer. Library construction proceeded with 200 ng of total RNA using the TruSeq Stranded Total Sample Preparation kit (Illumina) with Ribo-Zero ribosomal RNA depletion. WT and R2-10U virus sequencing for Fig. 3 and 5 was performed on a NovaSeq6000 sequencer (Illumina) using a Novaseq SP-100 flow cell (Illumina) generating 100 bp single-end reads, resulting in ~43 million reads per sample. WT, R2-10U, and R2-8U virus sequencing for Fig. 6 and 7 was performed on a NovaSeq X Plus sequencer (Illumina) generating paired-end reads, resulting in ~20 million reads per end. The obtained reads were aligned to the GRCh38 human reference genome using Bowtie 2 (version 2.2.9) (37). The unmapped reads were then applied to ViReMa (version 0.25) or VODKA to identify junction reads from cbDVGs according to previous publications (8, 38). For VODKA output, reads were additionally assessed using NCBI nucleotide BLAST, and junction positions were compared between the two. Any reads with different junction positions between the two algorithms were removed, and the sequences of filtered DVG junction reads were then obtained. To determine the number of consecutive As in the mutation region within DVG junction reads, we first selected DVG junction reads with rejoin point positions between nucleotides 15,044 and 15,098. Using the “stringr” package in R, DVG reads covering the mutation regions were extracted with the sequence pattern GTT[A]*(:::|TATTT), and the number of consecutive As was subsequently counted. The final cbDVG junction reads from the ViReMa output were used to graph the cbDVG break and rejoin point distribution in R. The reference RSV genome used for ViReMa and VODKA was the whole genome plasmid sequence of the RSV reverse genetic system backbone, pSynkRSV-line19F, or that containing the R2-10U or R2-8U mutations. To obtain the viral reads fully aligning to the viral reference genome, unmapped reads were aligned to the viral reference genome via Subread (39). Note that these viral reads included both non-cbDVG reads and reads aligned to regions of the genome homologous to cbDVGs (excluding the junction region) and are therefore considered as total viral reads. The coverage of viral reads for the whole viral genome, as well as mutation regions, was visualized and calculated using Integrative Genomics Viewer (IGV).

### RNA extraction and RT-qPCR for cbDVGs, viral genomes, and host/viral genes

Total RNA was extracted by TRIzol or TRIzol-LS according to the manufacturer’s specifications. To detect RSV cbDVGs, 1–2 μg of RNA was reverse transcribed using the Super Script III Reverse Transcription Kit (Invitrogen). RNA derived from minigenome experiments was treated with Turbo DNase prior to reverse transcription. The cbDVG RT-PCR screen and primers used have been previously described (8). Briefly, total RNA was reverse transcribed using the Super Script III Reverse Transcription Kit (Invitrogen). Reverse transcription of negative-sense cbDVGs 1563 and 1887 used primer 15292-F. qPCR amplification of both negative-sense cbDVGs used primers 15760-F and Small-Rev. Reverse transcription of positive-sense cbDVGs 1563 and 1887 used primer RSVDI1. qPCR amplification of positive-sense cbDVG 1563 used Small-Rev and 10UL1cbDVG1563-F, while positive-sense detection of cbDVG 1887 used Small-Rev and WTL2cbDVG1887-F. To quantify host/viral gene expression, total RNA was reverse transcribed using the High-Capacity RNA-to-cDNA Kit (Applied Biosystems). cDNA was diluted 1:20–1:40 and amplified with specific primers (Table S1) using the PowerTrack SYBR Green Master Mix (Applied Biosystems). qPCR reactions were performed in triplicate on a ViiA 7 Real-Time PCR System (Applied Biosystems). To quantify the RSV genome, total RNA was reverse transcribed using the Super Script III Reverse Transcription Kit (Invitrogen) using the gRSVDI-F primer. qPCR amplification of the genomic trailer region used gRSVDI-F and Small-Rev. All host/viral genes and cbDVGs were normalized to human GAPDH or hamster beta actin, depending on the cell line used. To quantify cbDVG pixel values, pixel densitometry analysis of agarose gel lanes from 100 to 600 bp, with background pixel values subtracted, was performed using ImageJ.

### Immunofluorescence

A549-RSV-N and the parental A549 cell line were seeded on cover slips and cultured overnight. Cells were washed 3× with PBS and fixed with 4% paraformaldehyde for 15 minutes at room temperature, followed by 3× PBS washes. Cells were permeabilized in 0.2% Triton X-100 for 10 minutes at room temperature. Cover slips were incubated with 2.5 μg/mL mouse anti-RSV N primary antibody (Abcam ab94806, 1 mg/mL) in PBS containing 3% FBS for 2 hours at room temperature. Cover slips were washed 3× with PBS and incubated with 2 μg/mL donkey anti-mouse Alexa Fluor 594 (Invitrogen A21203, 2 mg/mL) in PBS containing 3% FBS for 1 hour at room temperature. Cover slips were washed 3× with PBS, and cells were incubated with 1 μg/mL Hoechst (Invitrogen H3570, 10 mg/mL) in PBS for 7 minutes at room temperature. Cover slips were washed 3× with PBS and mounted on microscope slides using Fluoromount-G (Invitrogen 00-4958-02). Images were taken with a Leica DMIRB inverted fluorescence microscope with a cooled charge-coupled device (Cooke) on Image-Pro Plus software (Media Cybernetics). Images were color overlayed and compiled using ImageJ.

### Functional characterization of RSV cbDVGs

To determine the replication rates of cbDVGs, BSRT7 cells were co-transfected with cbDVG-expressing plasmids and plasmids expressing N, P, M2-1, and L. Cells were lysed with TRIzol at 2, 4, 6, 8, 12, and 24 hours post-transfection, and total RNA was extracted, Turbo DNase treated, and re-extracted. cbDVG-specific RT-qPCR was performed and quantified relative to hamster beta actin. To assess the host response to specific cbDVGs, cbDVG 1563- and cbDVG 1887-expressing plasmids were linearized with EcoRI, and 1 μg of linearized plasmid was used for *in vitro* transcription of cbDVG 1563 and cbDVG 1887 (Agilent RNAMaxx High Yield Transcription Kit) according to the manufacturer’s instructions. A549 or A549-RSV-N cells were transfected with 0.5–2 pmol of RNA for 6 hours at 37°C, followed by RNA extraction and examination of cbDVGs, IFN, and interferon-stimulated gene (ISG) expression by qPCR.

### *In vitro* transcription

A total of 10 μg of an RSV minigenome construct bearing the R2-10U mutation was linearized, and 275 ng of the linearized plasmid was *in vitro* transcribed (IVT) using the RNAMaxx High Yield Transcription Kit (Agilent) according to the manufacturer’s instructions, followed by lithium chloride precipitation. RNA sequencing was performed on the resulting IVT RNA, along with a previously generated virus stock, as described above.

### Statistical analysis

All statistical analyses were performed using GraphPad Prism version 10.0 (GraphPad Software). A statistically significant difference was defined as a *P*-value < 0.05 (based on specific statistical analyses as indicated in each figure legend).

## RESULTS

### R2-10U mutation altered trailer cbDVG rejoin point composition and attenuated cbDVG generation in an RSV minigenome system

**Fig 1:**
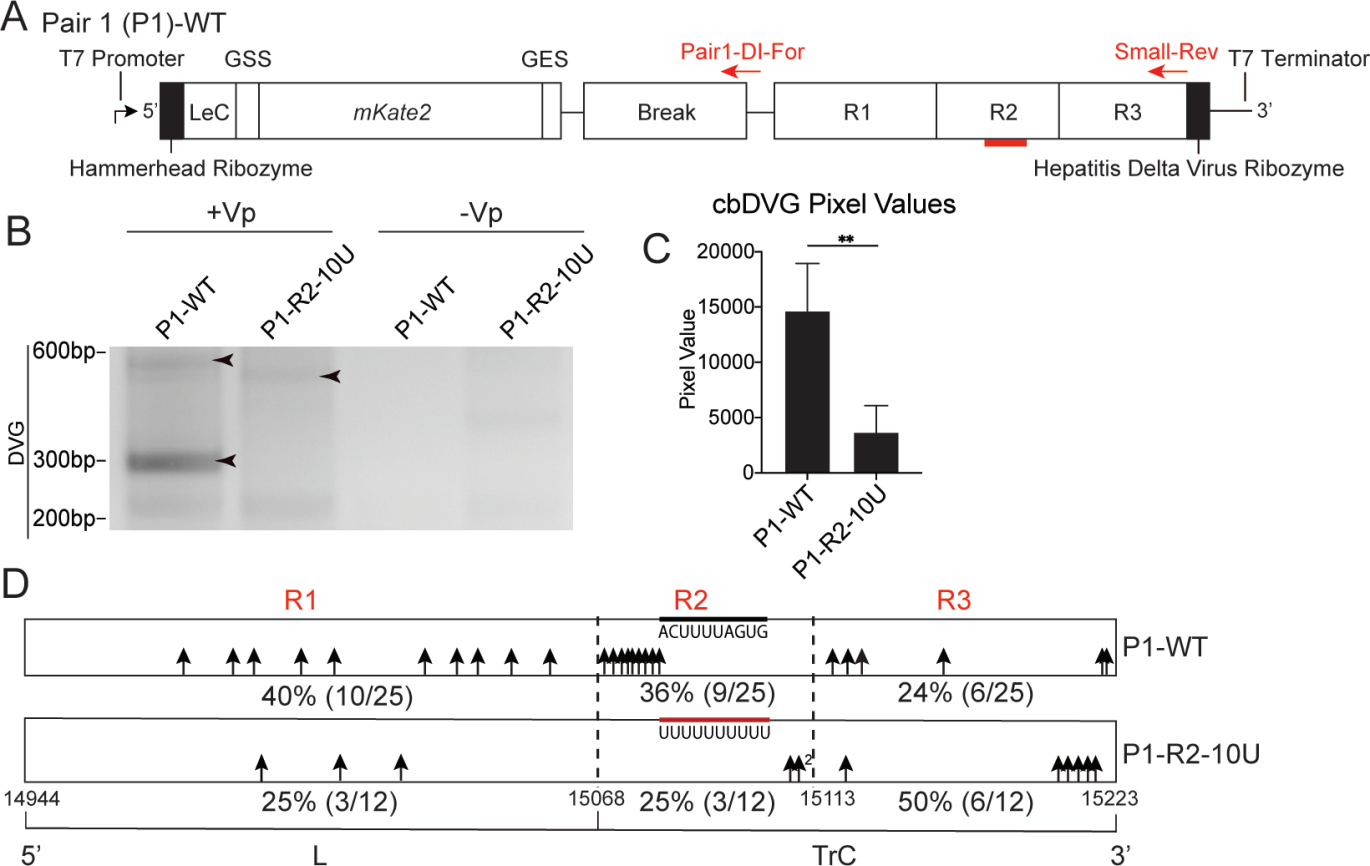
R2-10U mutation attenuated cbDVG rejoin point selection near and in the trailer region in an RSV minigenome system. (**A**) Schematic depiction of the P1-WT minigenome constructs. 5′–3′: T7 promoter, hammerhead ribozyme, leader complement sequence (LeC), L gene start signal (GSS), mKate2, L gene end signal (GES), Break, R1, R2, R3, hepatitis delta virus ribozyme, and T7 terminator. All minigenome constructs are encoded within the pSI1180 vector. The expression of mKate2 first requires synthesis of the negative-sense strand from the trailer to leader mediated by the viral proteins: N, P, M2-1, and L, encoded by the four co-transfected helper plasmids. cbDVG generation results from an aberrant round of genomic replication, which can be detected by DVG-specific RT-PCR. Bold red lines indicate the location of the 10U mutation within R2. Red arrows indicate the primers, and their locations, used for the cbDVG RT-PCR screen in panel B. (**B–D**) BSRT7 cells were co-transfected with four helper plasmids and P1-WT or P1-R2-10U, respectively, and cbDVG RT-PCR using Pair1-DI-For and Small-Rev was performed 48 hours post-transfection. (**B**) Representative agarose gel image of cbDVG-like amplicons detected by cbDVG RT-PCR screening. Black arrowheads indicate cbDVG-like amplicons verified by Sanger sequencing. The sequences of labeled cbDVG-like amplicons are compiled in Fig. S2. P1-WT and P1-R2-10U transfections lacking the helper plasmids (−Vp) were included as negative controls. (**C**) Pixel intensities of cbDVG-containing lanes were calculated by subtracting the gel background signal from cbDVG amplicon pixel values for each repeat. **P < 0.01 by paired *t* test, mean ± SD, *N* = 5. (**D**) Schematic summary of rejoin points (black arrows) of all sequence-verified cbDVGs from five biological repeats. Numbers immediately adjacent to the black arrows indicate the number of cbDVGs with the same rejoin point. Percentage of cbDVGs rejoined in each hotspot is shown beneath the scheme, with fractions of the total verified cbDVGs listed in parentheses.

Previously, we found that RSV cbDVG rejoin points clustered at the end of the L gene and within the viral trailer complement sequence (genomic positions 14944–15223) after cbDVG enrichment by high multiplicity of infection passaging, while break points were widely spread across the antigenome (8). To further characterize the antigenomic region clustered with cbDVG rejoin points, this region was divided into three hotspots with R1 at the end of the L gene, including the L gene end signal, and R2 and R3 in the trailer complement sequence (Fig. 1D). The introduction of a poly-U mutation into the R1 region of a recombinant RSV, mutating several key GC ribonucleotides to Us, reduced cbDVGs detected at R1 (8). Here, we sought to determine whether cbDVGs at R2 are also genetically manipulable by introducing a similar mutation in the R2 sequence. We first tested this using a previously developed RSV minigenome system. Briefly, we created a pair1 (P1)-WT construct, under the control of the T7 promoter, containing the RSV leader complement sequence (LeC), an mKate2 reporter gene, the sequence of one previously identified break hotspot (Break), and the wild-type rejoin hotspots R1–R3 (Fig. 1A). The P1-WT 5′ and 3′ genomic termini are flanked by the hammerhead and hepatitis delta virus ribozyme sequences, respectively, to generate precise template termini. The positive-sense P1-WT genome is first produced by the T7 polymerase. When co-transfected with helper plasmids encoding the viral factors required for replication and transcription (N, P, M2-1, and L), this RNA serves as the template for negative-sense strand synthesis, during which cbDVG generation predominantly occurs and can subsequently be detected by RT-PCR (8).

To test whether the proportion of cbDVGs at R2 is genetically manipulable, a span of 10 ribonucleotides (genomic positions 15095–15104) in R2 containing 3 total G/Cs was first mutated to 10 consecutive Us (R2-10U) in P1-WT (P1-R2-10U). BSRT7 cells were then transfected with helper plasmids and P1-WT or P1-R2-10U, and cells were collected for RNA extraction 48 hours post-transfection (hpt). Using cbDVG-specific RT-PCR, we consistently observed that cbDVG-like amplicons generated by P1-WT were of greater intensity compared to those generated by P1-R2-10U, none of which were detected in the absence of helper plasmids (Fig. 1B, primer locations indicated by red arrows in Fig. 1A). This observation was further confirmed by pixel density quantification of cbDVG-like amplicons (Fig. 1C). To assess how the R2-10U mutation impacts cbDVG rejoin point selection, cbDVG-like amplicons were cloned, and their break and rejoin points were determined by Sanger sequencing. For P1-WT, consistent with previous observations, 40% (10/25) of cbDVGs had rejoin points within R1, while 36% (9/25) and 24% (6/25) had rejoin points within R2 and R3, respectively. In contrast, P1-R2-10U had fewer total identified cbDVGs and reduced percentages of cbDVGs containing R1 or R2 rejoin points compared to P1-WT (Fig. 1D). To test the transfection efficiencies of P1-WT and P1-R2-10U plasmids, we co-transfected four helper plasmids, T7-GFP, with P1-WT or P1-R2-10U, and observed similar percentage of GFP-positive cells between P1-WT and P1-R2-10U (Fig. S1B). Moreover, by RT-qPCR, we detected more P1-R2-10U templates expressed by the T7 polymerase than P1-WT in the absence of helper plasmids, indicating that the fewer cbDVG-like amplicons detected from P1-R2-10U were not due to lesser transfection efficiency or RNA template production (Fig. S1C). Taken together, the lesser intensity of DVG amplicons, along with the reduced number of cbDVGs with rejoin points in R1–R3 (herein referred to as trailer cbDVGs) derived from P1-R2-10U, suggests that the R2-10U mutation may generally attenuate the generation and/or propagation of trailer cbDVGs.

### R2-10U mutation resulted in a delayed detection of trailer cbDVGs during infection

**Fig 2:**
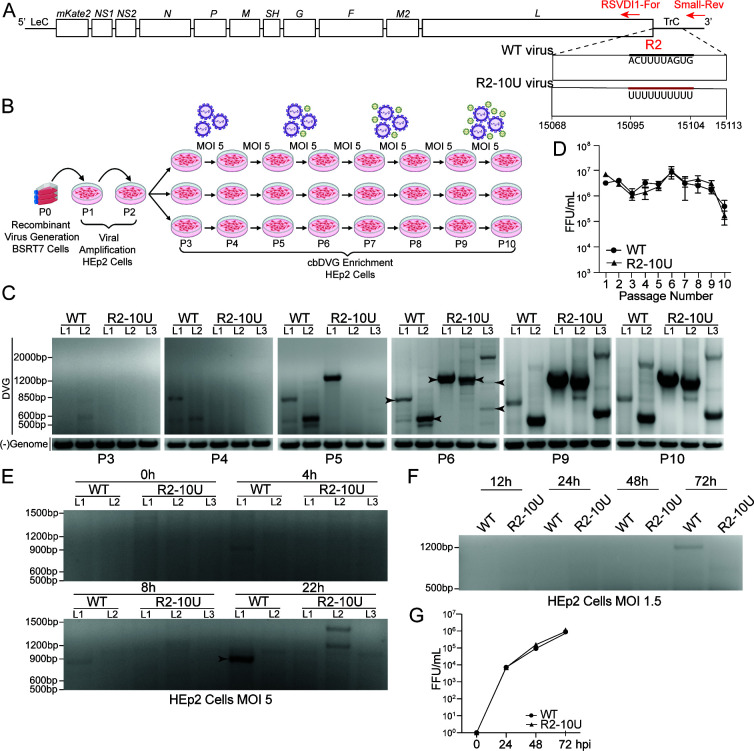
R2-10U mutation resulted in a delayed detection of trailer cbDVGs during infection. (**A**) Schematic of the recombinant WT and R2-10U viruses. Bold black line indicates the WT sequence, and bold red line indicates the R2-10U mutation at antigenomic positions 15095–15104. Red arrows indicate the primers, and their locations, used for cbDVG RT-PCR screening in panels C and E–G. (**B**) Depiction of high MOI cbDVG enrichment strategy. Recombinant viruses were rescued in BSRT7 cells at P0. Recombinant viruses were further amplified in HEp2 cells during P1 and P2. Starting at P3, two lineages of the WT virus and three lineages of the R2-10U virus were generated. P3–P10, HEp2 cells were infected at an MOI of 5 for 3 days per passage. Supernatants from each passage were titered and used to generate the subsequent passage. Large purple virus particle icons indicate infectious virus. Small green virus icons indicate defective interfering particles (**C**) Agarose gel images of cbDVG RT-PCR screens (top) and negative-sense genome RT-PCR (bottom) for P3–P6, P9, and P10 viruses. Lanes corresponding to each lineage for the WT and R2-10U viruses are indicated above. Arrowheads indicate Sanger sequencing confirmed cbDVG amplicons. (**D**) Virus titers were determined by fluorescent-forming units for each passage and indicated using FFU/mL. *N* = 2 WT lineages, *N* = 3 R2-10U lineages, mean ± SD. (**E**) Agarose gel image of cbDVG RT-PCR screen of HEp2 cells infected at an MOI of 5 with WT LD (L1–L2) or R2-10U LD virus (L1–L3). Arrowhead indicates Sanger sequencing confirmed cbDVG amplicon with sequences shown in Fig. S2. (**F and G**) Agarose gel image of cbDVG RT-PCR screen (**F**) of HEp2 cells infected at an MOI of 1.5 with the WT LD (L2) or R2-10U LD (L2) virus. Virus titers were determined at 24, 48, and 72 hpi from the same infection. Viral titration was performed in HEp2 cells (**G**).

To determine how the R2-10U mutation impacts trailer cbDVG production and propagation during viral infection, the R2-10U mutation was introduced into the RSV reverse genetic system (R2-10U virus, Fig. 2A) (40). Both R2-10U and WT recombinant viruses were successfully rescued and further propagated in HEp2 cells for two passages. To enrich trailer cbDVGs, both passage 2 (P2) viruses were serially passaged at a fixed MOI of 5 until passage 10 (P10). To generate technical replicates, beginning at P3, two separate sets of HEp2 cells were inoculated with the WT virus, while three separate sets of HEp2 cells were inoculated with the R2-10U virus (Fig. 2B). Supernatants and infected cells were collected and used to monitor virus titers and cbDVG emergence via FFU/mL determination and cbDVG-specific RT-PCR, at each passage, respectively. For cbDVG RT-PCR, primers specifically targeting trailer cbDVGs were used (indicated by red arrows in Fig. 2A). A cbDVG amplicon generated by the WT L2 virus was first detected at P3, similar to previous polyU mutations in R1 (8), and cbDVG amplicons by the WT L1 virus were first detected by P4. Interestingly, for the R2-10U viruses, cbDVG amplicons were first detected at P5 (L1) and P6 (L2 and L3), denoting a striking 2–3 passage delay in cbDVG detection compared to WT (Fig. 2C). No differences in virus titer were observed between the WT and R2-10U viruses between P1 and P10 (Fig. 2D). To verify this delay, we generated WT or R2-10U virus stocks containing low amounts of cbDVGs (LD) by serial passaging P2 at an MOI of 0.001 for three additional passages. HEp2 cells were then infected with these LD stocks at an MOI of 5 or 1.5. While high MOI infection encourages pre-existing cbDVGs that compete for efficient replication to further accumulate, lower MOI infection restricts such accumulation, thereby increasing the likelihood of detecting newly generated cbDVGs. As expected, we detected a verified cbDVG for the WT L1 virus as early as 4 hpi under the high MOI condition, while cbDVG amplicons were not detected until 22 hpi for the R2-10U virus (Fig. 2E). Given that minimal progeny RSV particles are released before 24 hpi, viral titer determination was not performed. Correspondingly, during the MOI 1.5 infections, a cbDVG amplicon was detected at 72 hpi during WT virus infection, while no cbDVG bands were detected during R2-10U virus infection at any timepoint assessed (Fig. 2F). No differences in virus titers were observed at any examined time point between the WT or R2-10U viruses (Fig. 2G). Altogether, these results suggest that the R2-10U virus reduced the generation and/or propagation of trailer cbDVGs compared to the WT virus during infection.

### R2-10U mutation diminished cbDVGs with rejoin points at R2 during viral infection

**Fig 3:**
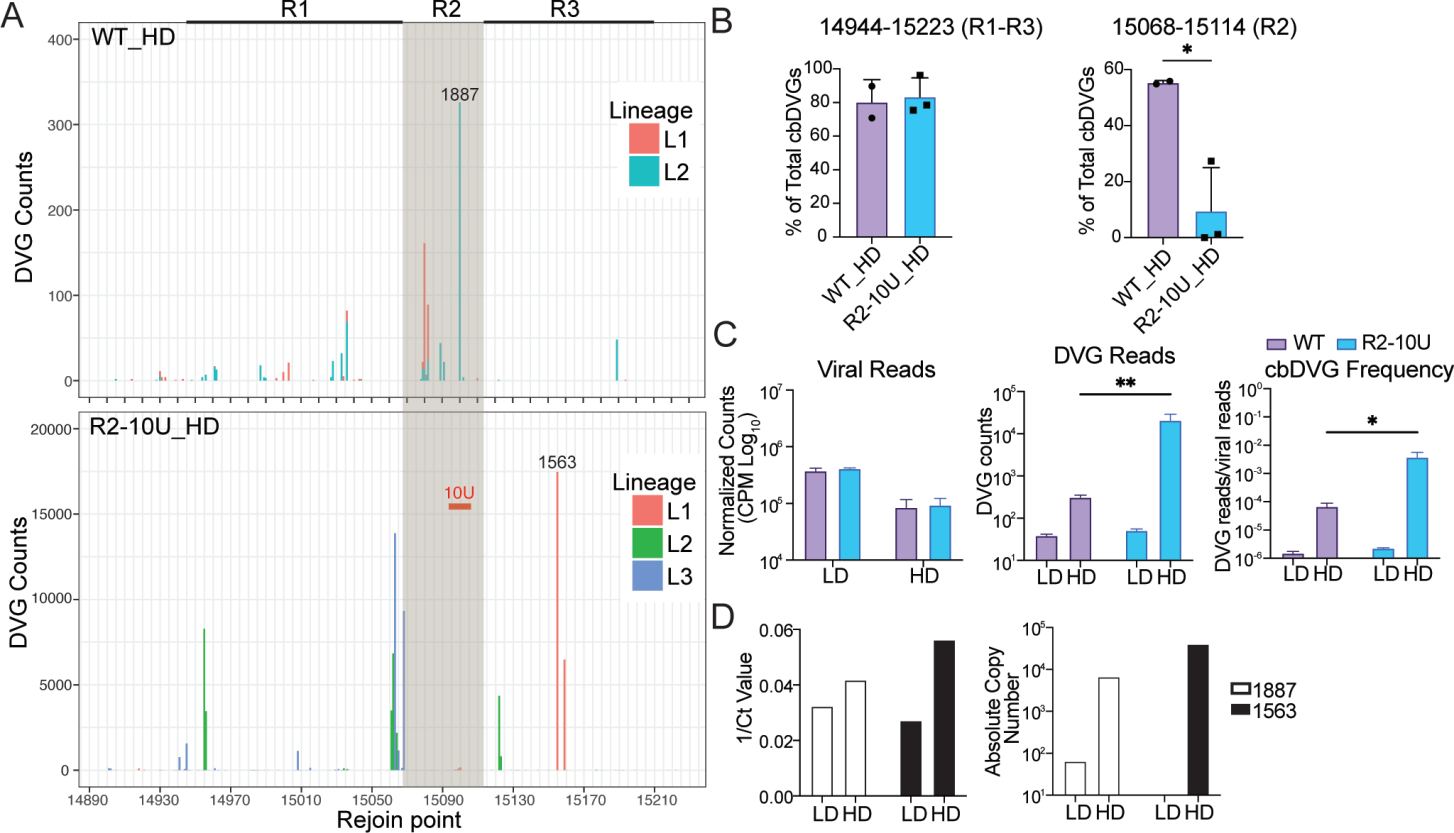
R2-10U mutation diminished cbDVGs with rejoin points at R2 during viral infection. Bulk RNA-seq was performed to analyze the WT and R2-10U P10 HD viruses. (**A**) Histograms depicting cbDVG rejoin point distributions of the WT and R2-10U viruses. The total number of P10 cbDVG rejoin points at each genomic position spanning 14890–15223 is illustrated. The shaded area indicates the location of the R2 region. The bold red bar within the shaded region indicates the location of the R2-10U mutation. The bold lines to the left and right of R2 indicate R1 and R3, respectively. cbDVG 1887 and 1563 were selected from the WT and R2-10U HD viruses, respectively, as the dominant cbDVG species for further verification, and their rejoin points are indicated on the rejoin point distribution histogram. (**B**) The percentage of trailer cbDVGs (left) or cbDVGs with rejoin points only at R2 (right) relative to the total cbDVGs was plotted for WT HD and R2-10U HD. *P < 0.05 by unpaired *t* test, mean ± SD. (**C**) Viral reads fully aligning to the RSV reverse genetic system backbone reference genome for the P10 HD and P5 LD of the WT and R2-10U viruses. The total viral reads (left) were normalized to the total sequencing reads shown as log_10_(CPM). Total abundance of cbDVG reads (middle) and cbDVG frequency (right) for the P10 HD and P5 LD WT and R2-10U viruses. cbDVG frequency was calculated by normalizing cbDVG reads to viral reads for each respective lineage of the WT and R2-10U viruses. (**D**) RT-qPCR validation of cbDVG 1887 and 1563 abundances within WT L2 and R2-10U L3 HD and LD virus supernatants, respectively.

To determine whether the R2-10U mutation impacts the composition of the trailer cbDVG population, total RNA was extracted from the high cbDVG content (HD) P10 viruses for bulk RNA sequencing. As a control, total RNA was also extracted from LD stocks of each WT and R2-10U virus lineage (P5, used in Fig. 2E through G). To identify cbDVG reads, the ViReMa algorithm was used (38). ViReMa identifies the unique break and rejoin junction sequences specific to cbDVGs that do not fully align with the viral reference genome. To assess how the R2-10U mutation impacted the rejoin point composition of the cbDVG population, the rejoin point distributions of cbDVGs spanning the entire genome (Fig. S3A) and specifically those within genomic positions 14890–15223 (Fig. 3A), containing R1–R3, were plotted for both the WT and R2-10U P10 HD viruses. Consistent with previous observations (8), trailer cbDVGs dominated the DVG population in the WT and R2-10U viruses following high MOI passaging (Fig. S3A). Specifically, R2 (54%) was the major rejoin hotspot for WT HD, followed by R1 (20.1%) and finally R3 (3.97%). For R2-10U HD, however, the frequency of cbDVGs containing R2 rejoin points was significantly reduced relative to R1 and R3 (Fig. 3B), indicating that R2 sequences, particularly those within the mutation region, play a key role in influencing the generation and/or propagation of trailer cbDVGs containing R2 rejoin points. Like previous results, cbDVG break point clusters were widely distributed across the antigenome ranging from genomic positions 6000 to 15000, and these clusters were not conserved among different lineages of the WT and R2-10U viruses (Fig. S3B).

Next, to determine the number of reads fully aligned to the viral reference genome (viral reads), the Subread aligner was used (39). To account for potential differences in sequencing depth, viral reads were normalized to total sequencing reads and shown as counts per million (cpm). As expected, viral reads were reduced for both the WT and R2-10U HD viruses compared to their respective LD viruses, indicating that replication-competent trailer cbDVGs were sufficiently enriched to reduce the proportion of their respective standard viruses (Fig. 3C). Interestingly, although the dominant trailer cbDVGs in R2-10U HD were detected at a later passage compared to WT HD (Fig. 2C), R2-10U-HD had significantly greater total cbDVG reads at P10. This increase remained when cbDVG reads were normalized to viral reads (cbDVG frequency) (Fig. 3C). To validate the difference in cbDVG abundance, we examined the absolute amounts of specific major cbDVG species in WT and R2-10U HD stocks via RT-qPCR. Several criteria were established to select the dominant cbDVG species from the WT and R2-10U HD viruses. First, cbDVGs comprising >15% of their respective lineage must have been confirmed by VODKA (Table 1), another algorithm identifying cbDVGs from deep sequencing data sets (8). Next, the selected cbDVGs must represent a similar percentage of their lineage according to both ViReMa and VODKA. Finally, for the convenience of primer design, the selected R2-10U and WT cbDVGs must contain similar break positions but rejoin points in different rejoin hotspots. Therefore, WT cbDVG 1887, rejoined in R2, and R2-10U cbDVG 1563, rejoined in R3, were selected, and specific primers for cbDVG RT-qPCR were designed (Fig. S3C). Consistent with the deep sequencing findings, 1563 exhibited greater 1/Ct and absolute copy number values compared to 1887 in their corresponding HD virus lineages (Fig. 3D, standard curves in Fig. S3D). Notably, the most abundant cbDVGs detected by ViReMa were also detected by VODKA, indicating an overall agreement between these algorithms and that no putative major cbDVGs were omitted (Table 1). Those few discrepancies, among the percentage of total cbDVGs, observed between these algorithms may be due to their respective computational approaches. While VODKA generates an index of all potential cbDVG junctions from a given reference, ViReMa iteratively aligns reads to the reference to identify junction-containing reads. Overall, these results demonstrated that the R2-10U mutation largely reduced cbDVGs with rejoin points at R2 in trailer cbDVG-enriched virus stocks.

**TABLE 1:** Compilation of major cbDVG species identified by ViReMa and VODKA^*a*^

Virus	Lineage	Break (genomic position)	Rejoin (genomic position)	Rejoin hotspot	ViReMa: counts	ViReMa: % of total	VODKA: counts	VODKA: % of total	Name (estimated DVG length)
**R2-10U**	**L1**	13728	15155	**R3**	23,951	**95**	17,189	**92.6**	**1563**
R2-10U	L2	12687	14955	R1	7,840	30.2	7,450	37	2804
R2-10U	L2	9600	14126	R1	4,070	15.7	3,737	18.6	6720
R2-10U	L2	11722	15063	R1	3,999	15.4	1,435	7.1	3661
R2-10U	L3	12138	15063	R1	7,543	66	409	6.9	3245
WT	L1	11669	15080	R2	169	49.4	155	24.6	3697
**WT**	**L2**	13469	15100	**R2**	**106**	**37.7**	**105**	**34.3**	**1887**

^*a*^ cbDVGs comprising >15% of the cbDVG population in P10 viruses according to ViReMa are indicated. VODKA identification of these cbDVGs and their percentage of the cbDVG population are shown. cbDVGs highlighted in bold indicate cbDVGs selected for functional analysis.

### R3 cbDVG 1563 has enhanced replication kinetics and similar immunostimulatory activity compared to R2 cbDVG 1887

**Fig 4:**
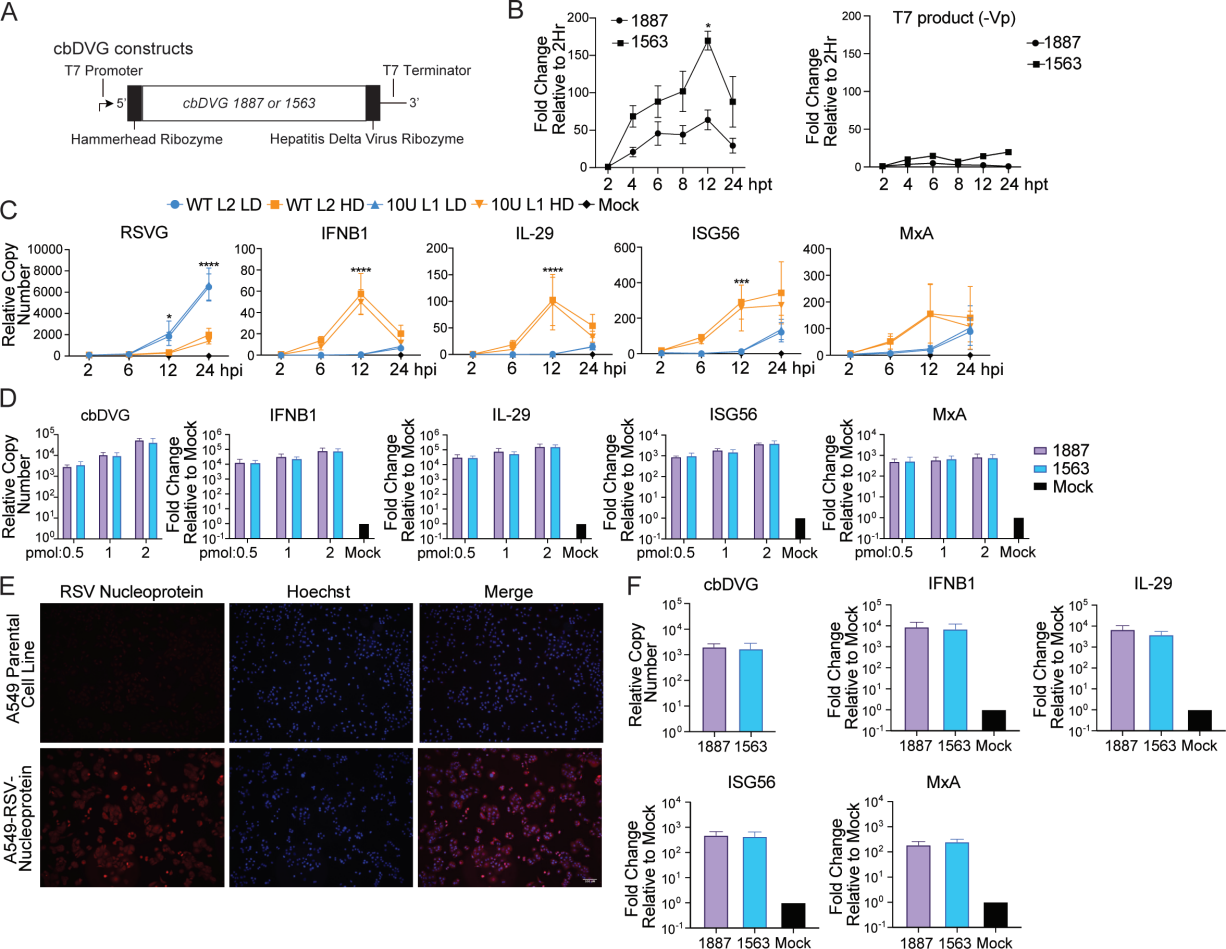
cbDVG 1563 has enhanced replication kinetics and similar immunostimulatory activity compared to cbDVG 1887. (**A**) Schematic depiction of select dominant cbDVG 1887- and 1563-expressing constructs. (**B**, left) BSRT7 cells were co-transfected with four helper plasmids and plasmids expressing cbDVGs 1887 or 1563, respectively, followed by primer-specific RT-qPCR (strategy illustrated in Fig. S3). cbDVG copy numbers were calculated relative to hamster beta actin, and background T7 product values were subtracted. Relative copy numbers were normalized to 2 hours post-transfection. *P < 0.05 by two-way ANOVA with Sidak’s multiple comparisons test. *N* = 3 independent repeats, mean ± SD. (**B**, right) BSRT7 cells were transfected with plasmids expressing cbDVG 1887 or 1563, and cells were collected at the same time points, followed by RT-qPCR targeting positive-sense DVGs (T7 products). Relative copy numbers were normalized to 2 hours post-transfection. (**C**) A549 cells were infected at an MOI of 1 with WT L2 HD, R2-10U L1 HD, WT L2 LD, or R2-10U L1 LD. The expression of IFNB1, IL-29, ISG56, MxA, and RSV G was examined via RT-qPCR at 2, 6, 12, and 24 hpi. Copy numbers were calculated relative to GAPDH. *P < 0.05, **P < 0.01, ***P < 0.001, and ****P < 0.0001 by mixed-effects analysis with Tukey’s multiple comparisons test for RSV G, IFNB1, IL-29, ISG56, and MxA. *N* = 3 independent repeats, mean ± SD. (**D**) cbDVG 1563 and 1887 were *in vitro* transcribed, and 0.5, 1, or 2 pmol of cbDVG RNA was transfected into A549 cells for 6 hours followed by RNA extraction. RT-qPCR targeting IFNB1, IL-29, ISG56, MxA, and cbDVG 1563 or 1887 was performed. Copy numbers were calculated relative to GAPDH, and relative copy numbers were then normalized to mock-transfected cells. *N* = 4 independent repeats, mean ± SD. (**E**) A549 cells were transduced to constitutively express the RSV nucleoprotein, followed by single clone propagation. Immunofluorescence images using an anti-RSV nucleoprotein antibody for parental and A549-RSV-N cells are shown. (**F**) A549-RSV-N cells were transfected with 0.5 pmol of cbDVG 1563 or 1887 for 6-hours followed by RNA extraction. RT-qPCR targeting IFNB1, IL-29, ISG56, MxA, and cbDVG 1563 or 1887 was performed. Copy numbers were calculated relative to GAPDH, and relative copy numbers of each gene were normalized to mock-transfected cells. *N* = 3 independent repeats, mean ± SD.

Our deep-sequencing data indicated that the dominant cbDVGs from the R2-10U viruses were enriched to a higher level compared to WT cbDVGs, despite their delayed detection. We hypothesized that this may be due to enhanced replication rates of those dominant cbDVGs enriched in the R2-10U HD viruses. To test this, we compared the replication rates of the two dominant cbDVGs previously selected from the WT (1887) and R2-10U (1563) P10 HD virus stocks. cbDVGs 1887 and 1563 were first cloned under the control of the T7 promoter. Specifically, the 5′ and 3′ ends of these cbDVG sequences were flanked by ribozyme sequences to express cbDVGs with accurate termini (Fig. 4A). cbDVG-expressing plasmids were transfected into BSRT7 cells along with helper plasmids, encoding the viral proteins required for genomic replication, and cells were collected at 2, 4, 6, 8, 12, and 24 hpt. To specifically assay for cbDVG replication driven by the viral polymerase and distinguish from the T7 product, qPCR primers targeting the negative sense of these cbDVGs were used. By normalizing each time point to 2 hpt, 1563 replicated to a higher relative amount than 1887 at each time point tested, with significant differences between them at 12 hpt (Fig. 4B, left). As a control, we also examined the rate of cbDVG templates (positive sense) produced by the T7 polymerase post-transfection in the absence of helper plasmids and observed minimal accumulation (Fig. 4B, right), confirming 1563’s greater replication rate compared to 1887.

We next sought to evaluate whether cbDVG populations of differing compositions have distinct IFN stimulation abilities. We first selected WT L2 HD (P10) and R2-10U L1 HD (P10), composed of cbDVGs with rejoin points primarily in R2 (54.4%) and R3 (95%), respectively, for infection in A549 cells at an MOI of 1. A549 cells were alternatively infected with WT LD or R2-10U LD to serve as negative controls. Expectedly, the RSV glycoprotein gene (RSV G) was expressed at higher levels in both LD viruses compared to their respective HD viruses, indicative of cbDVG-mediated attenuation of standard virus replication. The expression of IFNB1, IL-29, and ISG56 was significantly elevated at 12 hpi during HD virus infections compared to LD virus infections (Fig. 4C). Comparison of WT HD with R2-10U HD, however, did not reveal detectable differences in antiviral gene expression at any time point, despite greater cbDVG abundance in the R2-10U virus than WT virus (Fig. 4C). To further examine the IFN stimulation of specific dominant cbDVG species identified from the WT and R2-10U HD viruses, we *in vitro* transcribed cbDVG 1887 and 1563 RNA, and A549 cells were transfected with 0.5, 1, or 2 pmol. Cells were harvested at 6 hpt followed by RT-qPCR targeting antiviral gene expression and these specific cbDVGs. At each concentration, we detected similar relative copy numbers of 1887 and 1563, indicating similar transfection efficiencies. Relative to mock, at each respective picomolar amount, 1887 and 1563 induced similar expression of IFNB1, IL-29, ISG56, and MxA, indicating that 1887 and 1563 had similar immunostimulatory activities as naked RNAs (Fig. 4D). However, cbDVGs are generated during viral replication and therefore should be, at least partially, encapsidated with the RSV nucleoprotein during infection. Consequently, the presence of the nucleoprotein could impact the ability of a cellular RNA sensor to detect cbDVGs. Therefore, we generated an A549 cell line constitutively expressing the RSV nucleoprotein (A549-RSV-N, Fig. 4E). A549-RSV-N cells were transfected with 0.5 pmol of IVT cbDVG 1887 or 1563 RNA, and antiviral gene expression was assessed. Again, no differences were observed in the expression of IFNB1, IL-29, ISG56, or MxA genes between cbDVG 1887 and 1563 (Fig. 4F). Taken together, these data suggest that cbDVGs formed in R2 and R3 likely have similar abilities to induce antiviral signaling.

### A 2-ribonucleotide deletion emerged during R2-10U virus passaging

**Fig 5:**
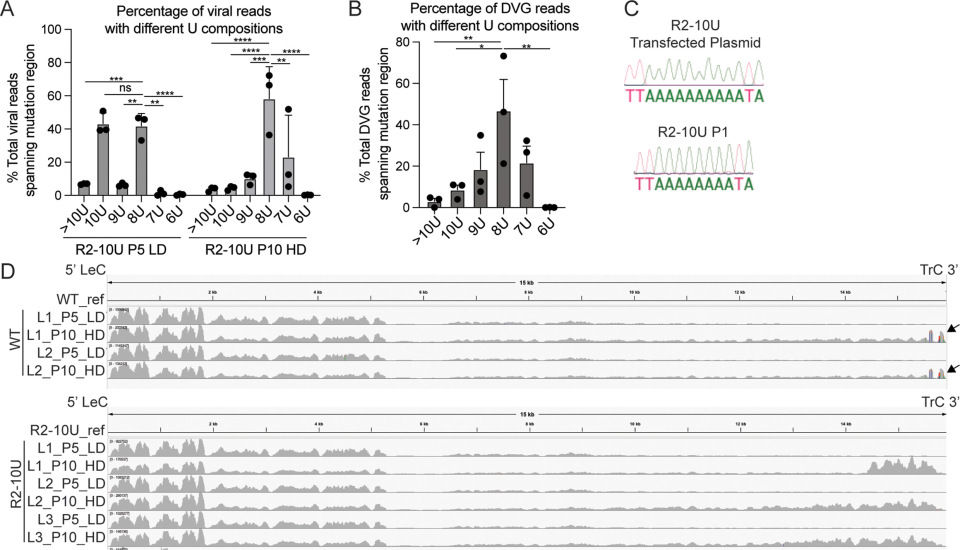
A 2-ribonucleotide deletion emerged in the R2-10U virus. (**A and B**) Among the viral reads (**A**) or DVG junction reads (**B**) that aligned to the R2-10U mutation region, the percentage of reads containing 6, 7, 8, 9, 10, or >10 Us was calculated for the R2-10U viruses. (**C**) Sanger sequencing identified the R2-8U mutation in the P1 R2-10U virus-infected cells (bottom) compared to the R2-10U mutation within the plasmid used to generate the recombinant virus via the RSV reverse genetic system (top). (**D**) RNA-seq reads from the WT and R2-10U viruses were aligned to the WT or R2-10U reference genome, respectively, via subread. The coverage of viral reads was graphed via IGV. Mutations accounting for more than 20% of total reads aligned to that position are colored. Arrows indicate the locations bearing the most variation in WT HD viruses compared to the WT reference genome. *P < 0.05, **P < 0.01, ***P < 0.001, and ****P < 0.0001 by two-way ANOVA.

To assess whether any mutations had arisen in the R2-10U mutation region during virus passaging, reads fully aligning to the R2-10U mutation were compared among lineages of the R2-10U P10-HD and P5-LD viruses. Interestingly, in addition to the original R2-10U sequence, in both P10 and P5, we detected various deletion mutations within the R2-10U sequence, including 1 (9U), 2 (8U), 3 (7U), and 4 (6U) ribonucleotide deletions. We calculated the percentage of reads containing a given deletion relative to the total number of reads aligning to the R2-10U sequence and found that R2-8U, a 2U-deletion variant, already accounted for more than 40% of the population in P5-LD stocks. In P10-HD, R2-8U accounted for, on average, 60% of the aligning reads, significantly more abundant than 10U and all other variants (Fig. 5A). Reads aligning to the mutation region could originate from either the full-length viral genome or the homologous regions of cbDVGs (distinct from the junction regions). Therefore, to specifically examine the percentage of R2-8U in the cbDVG population, DVG junction reads from P10-HD stocks were obtained via VODKA and filtered for those spanning the mutation region. Consistent with the viral read pattern, R2-8U constituted more than 60% of the total DVG reads (Fig. 5B). We additionally compared the proportion of 8U within the total viral reads versus within DVG junction reads and found no significant difference between the two. These results imply that R2-8U is the major variant in both full-length viral genomes and the DVG population. To assess when the R2-8U mutation may have arisen, we PCR amplified the viral genomic region containing R2-10U from earlier passages and identified R2-8U as early as P1 via Sanger sequencing, before the detection of cbDVGs by RT-PCR (Fig. 5C).

To examine mutations outside of the R2-10U region, we aligned RNA-seq reads to either the WT or R2-10U viral reference genomes and visualized aligned files with IGV (Fig. 5D) (41). Compared to LD, all HD stocks had additional coverage peaks at the 3′ end of the antigenome, indicating that those reads were from the homologous portions of cbDVGs when compared to the full-length viral genome. A similar observation was previously reported in Sendai virus HD stocks (42). We observed greater variation in the WT than the R2-10U viruses due to mutations identified at the 3′ antigenomic trailer of the WT HD stocks (arrows in Fig. 5D). Because of their specific locations, it is likely that these mutations were in trailer cbDVGs rather than full-length viral genomes. Interestingly, each of these mutations was A to G or U to C, consistent with the RNA editing pattern of adenosine deaminase acting on RNA enzymes and has been previously reported in cbDVGs of measles and human metapneumovirus (43, 44). The remaining mutations were considered minor, given that their WT ribonucleotide counterparts comprised more than 50% of the ribonucleotides at those positions (listed in Table S2).

### R2-10U virus had attenuated trailer cbDVG accumulation, whereas the R2-8U variant enhanced cbDVG dynamics compared to WT

**Fig 6:**
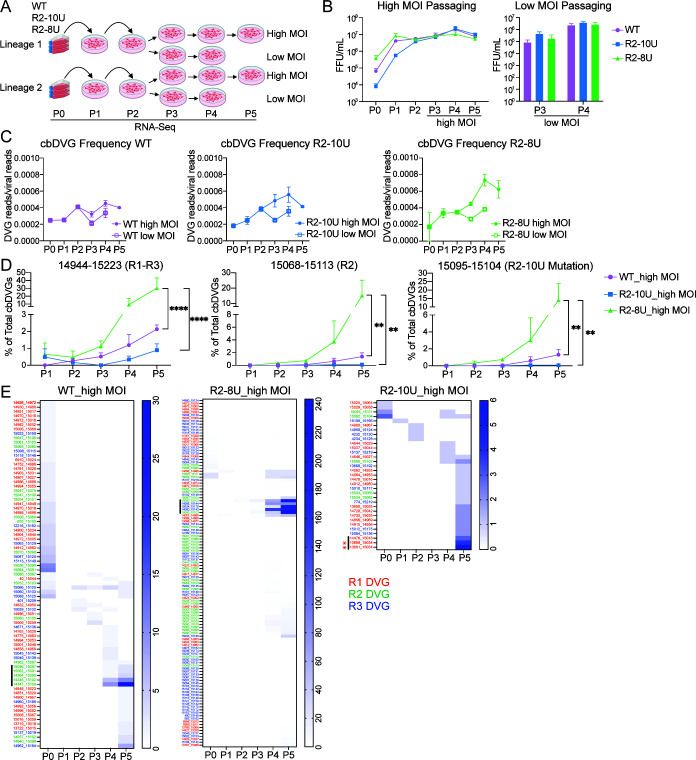
R2-10U virus had reduced trailer cbDVG generation, whereas R2-8U variant enhanced their dynamics compared to WT. (**A**) Schematic depiction of the strategy used for high or low MOI passaging of WT, R2-10U, and R2-8U viruses. P0 recombinant viruses, derived from two independent reverse genetic system transfections per virus, were rescued in BSRT7 cells. Recombinant viruses were further amplified in HEp2 cells during P1 and P2. Starting at P3, viruses were passaged either using an MOI of 5 or 0.001. Supernatants from each passage were titered and RNA sequenced. (**B**) Virus titers were determined by fluorescent-forming units for each passage either under an MOI of 5 (left) or 0.001 (right). (**C**) cbDVG frequencies were calculated by normalizing the total cbDVG reads to the total viral reads for each passage and virus under high MOI or low MOI passaging conditions. (**D**) Percentages of trailer cbDVGs (left), cbDVGs with rejoin points at R2 (middle), and cbDVGs with rejoin points in the mutation region (right) relative to the total cbDVG population in WT, R2-10U, and R2-8U viruses across passages. **P < 0.01 and ****P < 0.0001 by two-way ANOVA with Tukey’s multiple comparisons test. (**E**) Heatmaps of all trailer cbDVGs identified in each passage from WT, R2-10U, and R2-8U. Heatmap color intensity indicates the read abundance of each cbDVG obtained from VODKA. The numeric “break_rejoin” positions of each unique cbDVG are shown. Red, green, and blue colors indicate cbDVGs containing rejoin points within R1, R2, or R3, respectively. Bold black line indicates the major cbDVG species. *, indicates the major cbDVGs containing R2-8U in DVG junction reads.

Because R2-8U was the most frequent variant in the R2-10U virus, we sought to determine the impact of the R2-8U mutation on viral replication and trailer cbDVG dynamics. To do this, the R2-8U mutation was introduced into the RSV reverse genetic system to generate the recombinant R2-8U virus. For comparison, recombinant WT and R2-10U viruses were generated again in parallel, with each virus produced from two independent transfections of the RSV reverse genetic system. All viruses were then amplified and serially passaged in HEp2 cells at a fixed MOI of 5 (high MOI) or 0.001 (low MOI), similarly as before (Fig. 6A). Consistently, we found no significant differences in virus titers among WT, R2-10U, and R2-8U viruses at any tested passages (Fig. 6B). To assess the emergence and accumulation dynamics of trailer cbDVGs following serial passaging among different viruses, we deep sequenced total RNA from each passage, including P0. First, to examine the total amount of cbDVGs, we calculated the cbDVG frequency and found that high MOI passaging, in general, led to greater cbDVG accumulation relative to low MOI passaging (Fig. 6C). We then analyzed the distribution of cbDVG break and region point positions across the entire viral genome and observed that cbDVGs of large predicted sequence sizes with rejoin points near the leader end (genomic positions 0–4500) were more abundant than trailer cbDVGs in the early viral passages (Fig. S4A). A similar observation was previously reported by Felt et al. (45). Consistent with their report, we observed that trailer cbDVGs only accumulated following high MOI passaging (Fig. 6D versus Fig. S4B and C). Importantly, under high MOI passaging conditions, the rate of proportionate trailer cbDVG accumulation was least in the R2-10U virus, intermediate in the WT virus, and significantly greater in the R2-8U virus. This trend was particularly pronounced for cbDVGs with rejoin points specifically at R2 or within the mutation region (Fig. 6D). These results not only validated the delayed detection of trailer cbDVGs during R2-10U virus infection relative to WT by DVG-specific RT-PCR (Fig. 2 and 3) but also underscored the enhanced dynamics of trailer cbDVG during R2-8U infection. Finally, to determine the passages in which trailer cbDVGs that later dominate their respective cbDVG populations first emerged, we generated heatmaps of all identified trailer cbDVGs from each virus based on their abundance in each passage. The major trailer cbDVGs in P5-WT, P5-R2-8U, and P5-R2-10U stocks first appeared at P4, P3, and P5, respectively (indicated by the bold black line, Fig. 6E). Moreover, the R2-10U viruses displayed the fewest cbDVG species and the lowest abundance per cbDVG, whereas the R2-8U viruses showed the greatest number of cbDVGs and abundance per cbDVG (Fig. 6E). Taken together, these data indicate that the R2-10U virus contained few R2-derived cbDVGs and had delayed kinetics of trailer cbDVG accumulation, while its R2-8U variant reversed this phenotype, exhibiting an enhanced ability to produce and/or propagate trailer cbDVGs.

**Fig 7:**
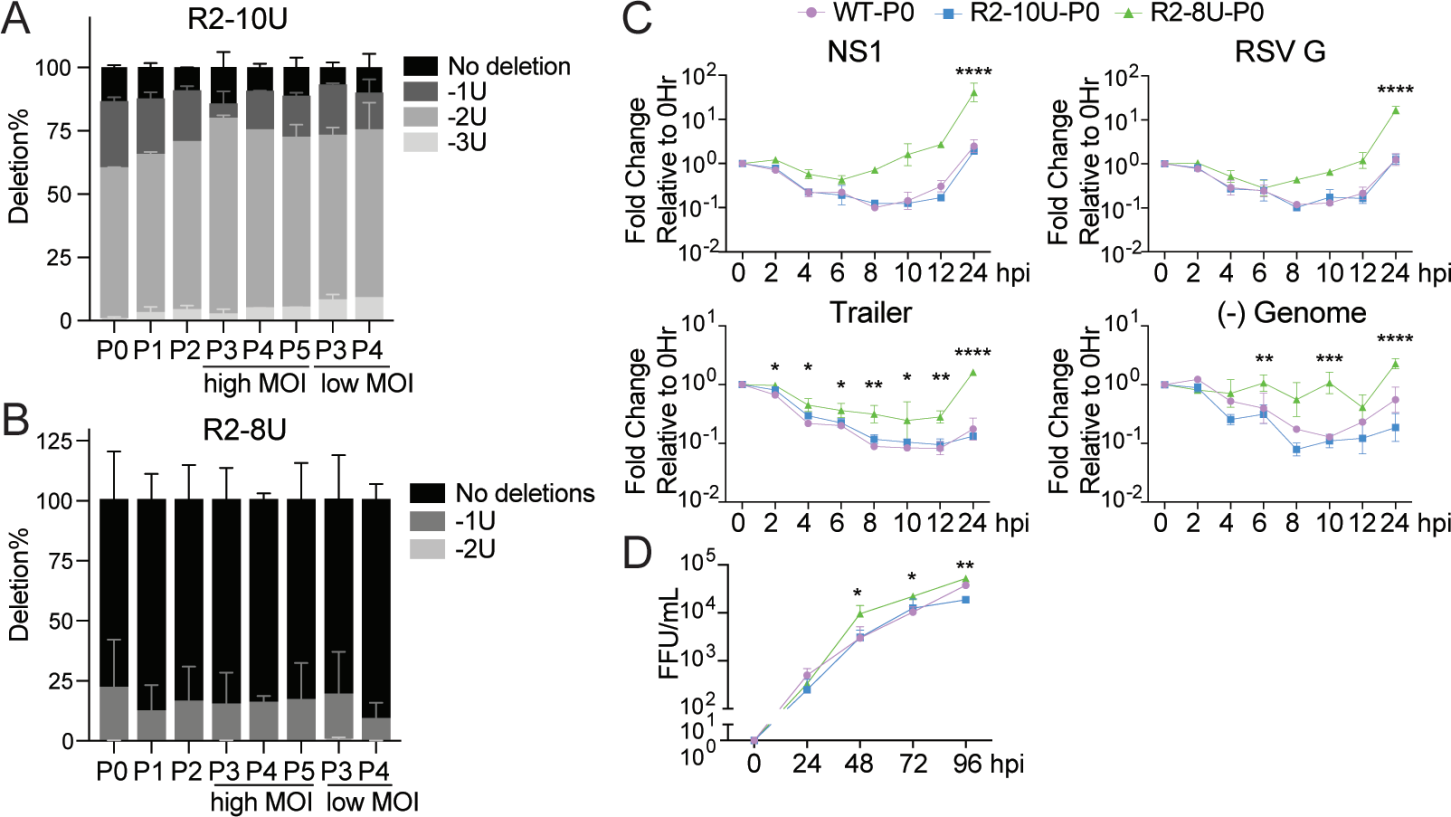
R2-8U has enhanced replication/transcription kinetics compared to R2-10U. (**A and B**) Among the viral reads from R2-10U (**A**) or R2-8U (**B**) that aligned to the R2-10U or R2-8U mutation region, the percentage of reads containing 7, 8, 9, or 10 Us was calculated for each passage. (**C**) BSRT7 cells were infected with WT, R2-10U, or R2-8U P0 viruses at an MOI of 0.0005. The expression of RSV NS1, G, trailer, and negative-sense genome was determined by RT-qPCR at 0, 2, 4, 6, 8, 10, 12, and 24 hpi. Copy numbers were calculated relative to hamster beta actin. Each time point was normalized to the 0 hpi relative copy number for each gene, respectively. (**D**) Progeny infectious virus production was determined by FFU/mL quantification at 24, 48, 72, and 96 hpi (bottom left). Viral titration was performed in BSRT7 cells. *N* = 2 biological repeats, mean ± SD. *P < 0.05, **P < 0.01, ***P < 0.001, and ****P < 0.0001 by two-way ANOVA analysis with Dunnett’s multiple comparisons test comparing R2-8U versus R2-10U.

Among the viral reads spanning the mutation region from the R2-10U virus, we again observed the R2-8U genotype as early as P0, consistently accounting for >60% of the total viral population across all passages, regardless of high or low MOI passaging conditions (Fig. 7A; Fig. S5A). In contrast, the R2-8U genotype remained relatively stable in the mutation region, with an average of 80% R2-8U (~20% 1U deletion variant) (Fig. 7B; Fig. S5B). Next, we similarly analyzed the DVG junction reads from the R2-10U virus following high MOI passaging. However, due to the limited number of trailer cbDVGs identified in R2-10U through P5, we identified only seven reliable DVG junction reads spanning the mutation region. Nonetheless, of these reads, four contained R2-8U, and one contained R2-10U. Notably, both of the two major trailer cbDVGs in R2-10U P5 carried R2-8U (marked with * in Fig. 6E). Short deletions can arise during sequencing of homopolymeric regions such as 10U. To specifically examine the error rate at the 10U sequence, we constructed an RSV minigenome backbone containing the 10U mutation under the T7 promoter to generate IVT RNA bearing the 10U mutation (Fig. S6A) and performed RNA sequencing using the same protocol. As an internal control, we also included RNA from one previously generated virus stock (R2-10U P5 under high MOI passaging, referred to as 10UP5Hi in Fig. S6). Interestingly, in the 10U IVT RNA sample, ~85% of reads were 10U, ~14% were 9U, and <1% were 8U, indicating a sequencing error rate of approximately 14% at the 10U region. However, this error is almost exclusively restricted to single-U deletions. In contrast, 10UP5Hi contained ~9% 10U, 13% 9U, ~75% 8U, and ~3% 7U reads, consistent with our previous results (Fig. S6B). Together, these sequencing data validate the emergence and dominance of the R2-8U variant in the R2-10U population and further suggest that the proportion of the 10U variant in the R2-10U population is likely underestimated due to sequencing errors (some 10U reads erroneously read as 9U). This may also account for the variation in 10U/9U abundance observed between Fig. 5A and 7A.

We next asked what drove the emergence of R2-8U during R2-10U virus infection and enabled the R2-8U genotype to more rapidly produce and propagate trailer cbDVG. We hypothesized that the R2-10U genotype negatively impacted genomic replication, leading to the emergence and selection of a variant with relatively enhanced genomic replication whose enhanced replication further accelerates cbDVG dynamics. Because R2-10U P0 contained 59.9% R2-8U, the least of any passage, P0 viruses were selected for low MOI growth curves. Additionally, because R2-8U emerged and accumulated in BSRT7 cells, BSRT7 cells were subsequently infected with WT, R2-10U, or R2-8U P0 viruses at an MOI of 0.0005. Infected cells were collected at 0, 2, 4, 6, 8, 10, 12, and 24 hpi, followed by RT-qPCR to examine viral replication and transcription levels. Relative to 0 hpi, the WT and R2-10U viruses exhibited comparable levels of RSV NS1, G, and the trailer region. In contrast, R2-8U demonstrated higher expression of these targets at every time point tested (Fig. 7C). RT-qPCR specifically targeting the negative-sense viral genome showed a similar trend (Fig. 7C), indicating that the R2-8U virus had enhanced replication/transcription activity compared to WT and R2-10U at the early stages of infection. Virus titers were additionally determined at D1, D2, D3, and D4 post-infection and showed that the R2-8U virus had a modest increase in virus titers compared to R2-10U at 48 hpi and each time point thereafter (Fig. 7D). It is worth noting that the average replication rate of the heterogeneous R2-10U virus population was consistently comparable to that of the WT virus by both RT-qPCR and multicycle growth curve analysis. In summary, these data indicate that R2 region mutations in the RSV trailer sequence impact early viral genomic replication, the kinetics of cbDVG emergence, and their subsequent accumulation dynamics.

## DISCUSSION

Historically, cbDVGs were considered a stochastic consequence of viral genomic replication driven by an error-prone RNA-dependent RNA polymerase exclusively during *in vitro* infection (46). However, several reports analyzing the cbDVG distribution profiles from different *mononegavirales* members observed that the rejoin points of enriched cbDVGs clustered at the 3′ end of the antigenome (7, 30, 31), arguing against the completely random generation theory. For RSV, we previously identified conserved cbDVG rejoin hotspots (R1–R3) at the end of the L gene and within the 3′ trailer of the RSV antigenome after high MOI passaging. Here, we refer to cbDVGs containing a rejoin point within R1–R3 as trailer cbDVGs. We demonstrated that mutating key G/C ribonucleotides at R1 can reduce trailer cbDVGs detected in R1 (8). Importantly, recent work has begun exploring the evolutionary and experimental factors impacting cbDVG dynamics and population composition. For example, Felt et al. (45) observed that cbDVGs of a large predicted sequence length, with break and rejoin points proximal to the 3′ leader end of the genome, predominate in early passages. In conditions favoring low levels of viral replication, such as low MOI passaging, these large cbDVGs are maintained but do not accumulate to a significant degree. However, sustained high levels of viral replication, such as high MOI passaging or infection in STAT1 KO cells, promote the diversification of the cbDVG population and emergence and accumulation of trailer cbDVGs (45). Consistent with this report, we observed that these large cbDVGs did not accumulate regardless of MOI and that the accumulation of trailer cbDVGs only occurred in the high MOI passaging condition. Additionally, the enhanced replication kinetics of R2-8U were associated with the faster accumulation of trailer cbDVGs in earlier passages, compared to WT and R2-10U. Overall, our observations support the notion that variables promoting enhanced levels of viral replication promote trailer cbDVG emergence and accumulation.

Recent evidence indicates that DVGs are not only generated in patients but also that DVGs and their kinetics play key roles in mediating infection outcomes (23, 25). For example, trailer cbDVGs have been detected by RNA-seq analysis of nasal samples from hospitalized RSV-positive pediatric patients (8). Likewise, recent deep sequencing of brain autopsy samples derived from an individual with subacute sclerosing panencephalitis, a progressive neurological disease caused by a persistent measles virus infection, detected numerous cbDVG species with rejoin points clustering at the trailer end of the viral genome (22). While the functions of trailer cbDVGs during human infection remain to be determined, these data highlight the clinical relevance of these cbDVGs. Here, we focused on trailer cbDVGs and showed that cbDVG generation at R2 was largely attenuated by mutating a sequence containing G and C ribonucleotides to 10 consecutive Us (R2-10U) within the R2 hotspot via both DVG-specific RT-PCR and unbiased deep sequencing. Furthermore, such attenuation was associated with delayed trailer cbDVG dynamics during R2-10U virus infection compared to WT (Fig. 6). Interestingly, we identified a 2-ribonucleotide deletion variant (R2-8U) that emerged as early as P0 within the R2-10U recombinant virus population. By deep sequencing analyses of the recombinant R2-8U virus, we observed that it not only recovered trailer cbDVG generation and propagation kinetics compared to the heterogeneous R2-10U population but also accelerated cbDVG dynamics compared to WT. This work provides evidence that the kinetics of trailer cbDVG emergence, in addition to their population composition, can be manipulated by mutating key sequences in rejoin hotspots. Although unstable, the recombinant R2-10U virus and its delayed cbDVG detection kinetics provides an opportunity to evaluate how delayed trailer cbDVG dynamics impact the host response and infection outcomes *in vivo*. Likewise, R2-8U provides an opportunity to evaluate how accelerated trailer cbDVG dynamics impact host responses and viral pathogenicity.

Because R2-8U emerged before trailer cbDVGs accumulated to a level sufficient for detection and remained the major viral genotype regardless of the content of trailer cbDVGs, we hypothesized that the emergence and dominance of the R2-8U variant was due to greater genomic replication efficiency than R2-10U. In support, the heterogeneous P0 R2-10U population, composed of approximately 60% 8U and 40% 10U/9U, replicated at a rate similar to WT but significantly lower than the R2-8U population, consisting of approximately 80% 8U and 20% 7U. Furthermore, we obtained two independent R2-8U lineages: the first contained 98.7% 8U, while the second consisted of ~60% 8U and ~40% 7U. These proportions remained stable across all passages (Fig. S5B). The replication kinetics of these R2-8U P0 populations were similar, and both replicated significantly faster than WT, demonstrating that R2-8U/7U variants possess greater replication efficiency than WT. Given these observations, it is likely that the heterogeneous R2-10U population displays replication kinetics similar to WT. While the 10U/9U variants replicate less efficiently than WT, the R2-8U/7U variants compensate for this defect, resulting in an average replication rate similar to that of WT.

While it is technically challenging to distinguish *de novo* cbDVG generation from their subsequent accumulation, our data strongly suggest that cbDVG generation at the R2 region was largely attenuated by the R2-10U mutation. For example, because cbDVG accumulation is a function of multiple rounds of the virus life cycle, given that the RSV minigenome system does not produce progeny infectious virus particles, it is likely that the cbDVG population observed in minigenome experiments is more representative of the *de novo* cbDVG population rather than enriched populations. Correspondingly, relative to the WT minigenome, we detected fewer cbDVGs containing R2 rejoin points, none within the R2-10U mutation sequence (Fig. 1). cbDVG detection in the minigenome system, however, is limited by PCR primer bias and greater sub-cloning efficiency of more intense agarose gel amplicons. Therefore, we further examined the kinetics of trailer cbDVGs from P0–P5 viruses by deep sequencing. Consistently, we detected few cbDVGs with rejoin points in R1–R3, or the mutation region, in the heterogeneous R2-10U population (Fig. 6). Furthermore, the two major trailer cbDVGs (rejoin points in R1) detected in R2-10U P5 contained the R2-8U, rather than the R2-10U, mutation. In contrast, the relatively homogeneous R2-8U virus had the earliest emergence of cbDVGs with rejoin points in R1–R3 and specifically within the R2 mutation region. Altogether, these data strongly suggest that the R2-10U mutation attenuates the generation of trailer cbDVGs, particularly R2 cbDVGs, while the R2-8U mutation restores it. The accelerated trailer cbDVG dynamics of R2-8U are likely driven by its enhanced genomic replication compared to R2-10U.

Despite R2-8U variant comprising 60%–80% of the R2-10U virus population, we observed distinct cbDVG dynamics in the heterogeneous R2-10U population compared to the relatively homogeneous R2-8U virus. We hypothesize that although 8U variants within the heterogeneous R2-10U population are capable of producing trailer cbDVGs, they must reach and maintain a sufficient proportion of the overall population for their derived cbDVGs to be stably detected during high-MOI passaging, as shown in Fig. 2. In support, the proportion of R2-8U variant within the R2-10U population steadily increased from P0 to P3 and remained between 70% and 80% thereafter (Fig. 7A). In contrast, R2-8U viruses already contained ~80% 8U at P0, and this ratio remained stable throughout passaging. Furthermore, the leftover ~20% 7U variant replicates efficiently and is likely to generate trailer cbDVGs as efficiently as 8U and therefore would not lead to the delayed cbDVG phenotype observed in R2-10U/9U. In Fig. 6E, in the R2-10U population, we observed five R2 cbDVGs (highlighted in green in Fig. 6E); however, these cbDVGs were not detected in subsequent passages or were at levels lower than R1 cbDVGs at P5. These R2 cbDVGs likely originated from R2-8U within the heterogeneous R2-10U virus population but were likely outcompeted by other trailer cbDVGs under high-MOI conditions. This observation was consistent with our deep-sequencing data from R2-10U P10 HD (Fig. 3), in which the frequency of R2 cbDVGs was markedly reduced compared to WT (Fig. 3A), whereas R1 and R3 cbDVGs were enriched by P10.

The terminal 36 nucleotides of the RSV trailer complement sequence constitute the antigenomic core promoter sequence, essential for viral genomic replication (47). Deletion mutagenesis experiments have demonstrated that RSV minigenome constructs and recombinant viruses containing trailer sequence lengths greater than the minimal trailer promoter sequence had more efficient promoter activity than the core promoter alone, indicating that a complete trailer complement sequence has inherently greater promoter activity (48, 49). To our knowledge, the R2-10U mutation is the first evidence that the trailer sequence composition of a full-length trailer, outside of the core promoter, impacts RSV genomic replication. More interestingly, the 2U deletion in this sequence significantly enhanced genomic replication. It has been hypothesized that the trailer sequences beyond the core promoter may support viral polymerase recruitment, polymerase transition to a stable elongation mode, or encapsidation (48). The precise replicative defect and augmentation driven by R2-10U and R2-8U, respectively, remain unclear.

It is worth noting that authentic cbDVG species generated during infection are di/triphosphorylated at their 5′ termini. Given their complementary ends, cbDVG species theoretically form a stem-loop structure with dsRNA ends in the absence of the nucleoprotein, forming the canonical RIG-I ligand (50). Although the cleavage of ribozyme sequences at both termini theoretically yields cbDVGs with accurate terminal ribonucleotides, cleavage results in cbDVGs lacking a 5′ di/triphosphorylated terminus. Additionally, unless the desired IVT products are specifically purified, the ribozyme cleavage products inherently remain. LiCl purification post-IVT limits the precipitation of small RNA ribozyme cleavage products; thus, it is unlikely that the ribozyme cleavage products comprised a significant proportion of the RNA species derived from our cbDVG IVT reactions (Bio-Rad). Previous reports have implicated the 5′ di/triphosphoryl group as a key component of cbDVG recognition by dsRNA sensors and antiviral signaling (51, 52). Although not directly assayed for, our data indicate that cbDVG 1887 and 1563 strongly induced the antiviral response in the absence of 5′ di/triphosphorylated termini. Nonetheless, these data indicate that, in the context of transfection-based experiments, cbDVG 1887 and 1563 have similar IFN and ISG induction potentials regardless of the presence or absence of the RSV nucleoprotein, suggesting that their IFN-stimulating motifs are likely in regions common between 1887 and 1563. More studies are needed to further verify this observation during infection by, for example, packaging specific cbDVGs into virus-like particles and supplementing them during LD virus infection.

The demonstration that RSV cbDVG populations are genetically manipulable provided a new avenue of investigation to assess many questions regarding the mechanisms of cbDVG generation and the impact of distinct cbDVG populations on RSV pathogenesis (8). In this study, we provide additional evidence that cbDVG generation can be genetically manipulated and demonstrate that the kinetics of cbDVG generation can be genetically manipulated as well. The R2-10U and R2-8U viruses provide novel tools to assess how cbDVG dynamics affect host responses. Furthermore, we observed that the R2-8U variant had enhanced genomic replication with a modest increase in virus titers, compared to its R2-10U progenitor and the WT virus, and accelerated trailer cbDVG emergence and accumulation kinetics. These observations identify a sequence in the RSV trailer region that, when mutated, critically modulated both viral replication and trailer cbDVG generation and propagation. They further suggest that cbDVG, particularly trailer cbDVG, generation may be an evolutionary tradeoff for more rapid viral replication kinetics. Overall, this work expands the repertoire of RSV genetic tools to alter cbDVG composition and kinetics, providing a unique platform to study RSV genomic replication, cbDVG-driven pathogenesis, and the evolutionary significance of cbDVGs.

## Data Availability

Deep sequencing data from passaging experiments are deposited at GEO (GSE281185).

## SUPPLEMENTAL MATERIAL

Supplemental material jvi.00184-26-s0001.pdf Figures S1 to S6; Tables S1 and S2.

ASM does not own the copyrights to Supplemental Material that may be linked to, or accessed through, an article. The authors have granted ASM a non-exclusive, world-wide license to publish the Supplemental Material files. Please contact the corresponding author directly for reuse.
