# Supplementary material for: A respiratory syncytial virus trailer sequence modulates viral replication and the generation and propagation kinetics of copy-back defective viral genomes
Source: J Virol. 2026 Mar 24;100(4):e00184-26. doi: 10.1128/jvi.00184-26 (PMC13098259; doi:10.1128/jvi.00184-26)
Supplement: Supplemental material — Figures S1 to S6; Tables S1 and S2. [file jvi.00184-26-s0001.pdf]

Supplementary Figures and Tables

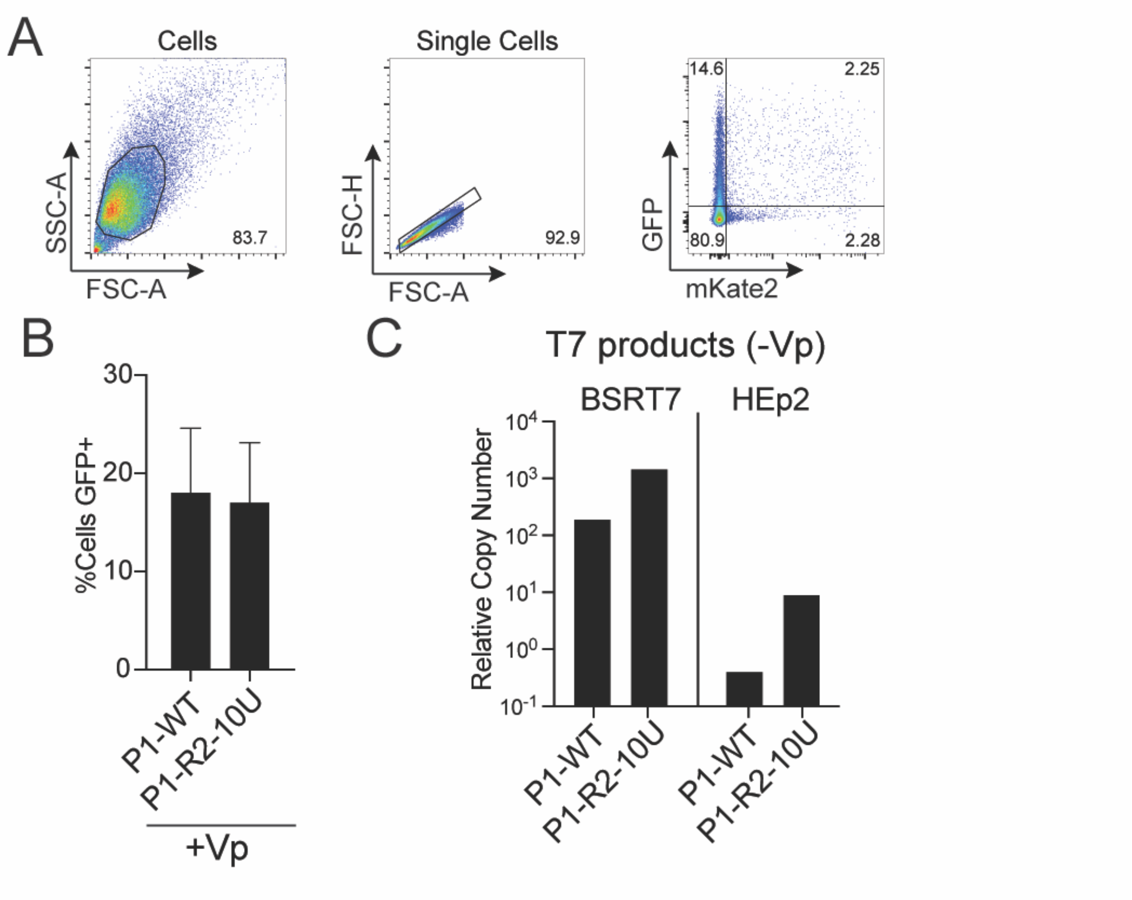

Figure S1: P1 minigenome constructs have similar transfection efficiencies. (A) representative gating strategy used for flow cytometry data. (B) BSRT7 cells were co-transfected with four helper plasmids encoding the viral components required for genomic replication, pcDNA3.1-EGFP and P1-WT or P1-R2-10U. EGFP expression in the presence of different minigenome constructs was measured by flow cytometry. N = 3 independent repeats, mean±SD. (C). BSRT7 (left) and HEp2 cells (right) were transfected with P1-WT or P1-R2-10U in the absence of the viral proteins required for genomic replication (-Vp). HEp2 cells served as a negative control for gene expression driven by the T7 polymerase. Expression of the minigenome T7 products were measured by qRT-PCR. N=1 independent repeat.

Fig1: P1-WT 600Bp
*CACCTCCGATCCCTTTAACTTA*CTGCCTAAGCATACATAAGTTTTTAATATAGTTATATTGTCTAATTTGAAATCAATATCATCTTGAGC
ATGATATTTTACTATTAACATACATTTATTAACTGAGGAACAGTACTTGCACTTTCTTACATGCTTGCTCCATTCTATTATAATTTTACTC
CAGTTGACTGTTACAGACAATTCGCGGCCGCTTTTTAATAACTATAATTGAATACTCATCTGTGCCCCAGTTTGCTAGGGAGGTCGC
AGTATCTGGCCACAGCCACCTCGTGCTGCTCGACGTAGGTCTCTTTGTCGGCCTCCTTGATTCTTTCCAGTCTTCTGTCCACATAG
TAGACGCCGGGCATCTTGAGGTTCTTAGCGGGTTTCTTGGATCTGTATGTGGTCTTCAAGTTGCAGATCAGGTGGCCCCCGCCCA
CGAGCTTCAGGGCCATGTCGGCTCTGCCTTCCAGGCCGCCGTCAGCGGGGTACAGGGTCTCGGTGGAGGCCTCCCAGCCGAG
TGTTTTCTTCTGCATCACAGGGCC:::CGA*GATATTAGTTTTTGACACTTTTTTTCTCG*

Fig1: P1-WT 300Bp
*CACCTCCGATCCCTTTAACTTA*CTGCCTAAGCATACATAAGTTTTTAATATAGTTATATTGTCTAATTTGAAATCAATATCATCTTGAGC
ATGATATTTTACTATTAACATACATTTATTAACTGAGGAACAGTACTTGCACTTTCTTACATGCTTGCTCCATTCTATTATAATTTTACTC
CAGTTGACTGTTACAGACAATTCGCGGCCG:::TATCATTTTAATCTTGGAGGAATAAATTTGAACCCTAATCTAATTGGTTTATATGT
GTATTAACTAAATTACGA*GATATTAGTTTTTGACACTTTTTTTCTCG*

Fig1: P1-R2-10U 500Bp
*CACCTCCGATCCCTTTAACTTA*CTGCCTAAGCATACATAAGTTTTTAATATAGTTATATTGTCTAATTTGAAATCAATATCATCTTGAG
CATGATATTTTACTATTAACATACATTTATTAACTGAGGAACAGTACTTGCACTTTCTTACATGCTTGCTCCATTCTATTATAATTTTAC
TCCAGTTGACTGTTACAGACAATTCGCGGCCGCTTTTTAATAACTATAATTGAATACTCATCTGTGCCCCAGTTTGCTAGGGAGGT
CGCAGTATCTGGCCACAGCCACCTCGTGCTGCTCGACGTAGGTCTCTTTGTCGGCCTCCTTGATTCTTTCCAGTCTTCTGTCCA
CATAGTAGACGCCGGGCATCTTGAGGTTCTTAGCGGGTTTCTTGGATCTGTATGTGGTCTTCAAGTTGCAGATCAGGTGGCCCC
CGCCCACGAGCTTCAGGGCCATGTCGGCTCTGCCTTCCAGGCCGCCGTCAGCGGGGTA:::TTAACTAAATTACGA*GATATTAGTT*
TTTGACACTTTTTTTCTCG

Fig2C: WT L2 600Bp
*CTTAGGTAAGGATATGTAGATTCTACC*ATATATAAATGGTTATAGTTTAGTTCTGTTGATCTGAAATTTAAAACATGATTGAACCATTTTA
AGATGTTCATATGCTTATGATTTATAAGTTTATTGCTGAAAACTTCATTACGTCCAGCTATAGAATATGATAGTATATCTCCACTAACAAC
ACTCTTTAGTTTTGACAATGCAGTATTAATTCCTTTTTTTGTTATAGGGTAACAAAGAAAGGGTATCAAACTTTTAATATTTGCATCAAT
AGACTCTTTATCAGCTTTCTTAGGCATGATGAAATTTTTGGTTCTTGATAGTATCAATTTAGCATTTTGTACTACATTAAATACTGGGA
ATATATTCGCAGGACCTATTGTAAGGACTAAGTAAACCTCCGATCCCTTTAACTTACTGCCTAAGCATACATAAGTTTTTAATATAGTT
ATATTGTCTAATTTGAAATCAATATCATCTTGAGCATGATATTTTACTATTAACATACATTTATTAACTGAGGAACAGTACTTGCACTTT
CTTACATGCTTGCTCCATTCTA:::CGA*GATATTAGTTTTTGACACTTTTTTTCTCG*

Fig2C: WT L1 850bp
*CTTAGGTAAGGATATGTAGATTCTACC*ATATATAAATGGTTATAGTTTAGTTCTGTTGATCTGAAATTTAAAACATGATTGAACCATTTT
AAGATGTTCATATGCTTATGATTTATAAGTTTATTGCTGAAAACTTCATTACGTCCAGCTATAGAATATGATAGTATATCTCCACTAACA
ACACTCTTTAGTTTTGACAATGCAGTATTAATTCCTTTTTTTGTTATAGGGTAACAAAGGAAGGGTATCAAACTTTTAATATTTGCATC
AATAGACTCTTTATCAGCTTTCTTAGGCATGATGAAATTTTTGGTTCTTGATAGTATCAATTTAGCATTTTGTACTACATTAAATACTGG
GAATATATTCGCAGGACCTATTGTAAGGACTAAGTAAACCTCCGATCCCTTTAACTTACTGCCTAAGCATACATAAGTTTTTAATATAG
TTATATTGTCTAATTTGAAATCAATATCATCTTGAGCATGATATTTTACTATTAACATACATTTATTAACTGAGGAACAGTACTTGCACTT
TCTTACATGCTTGCTCCATTCTATTATAATTTTACTCCAGTTGACTGTTACAGACAATTCGGCATCACAGACAAAAAGACTGATAGGT
TCAGCAAACTTTATATGTAAATAAGACCAATGAATGTTGTTGGTTGCATCTGTAGCAGGAATGGTCAAATTTTCACCATAA:::ACGATT
TTTTAAATAACTTTTAGTGAACTAATCCTAAAGTTATCATTTTAATCTTGGAGGAATAAATTTAAACCCTAATCTAATTGGT
TTATATGTGTATTAACTAAATTACGA*GATATTAGTTTTTGACACTTTTTTTCTCG*

Fig2C: R2-10U L1 1250Bp
ATTGTAAGGACTAAGTAAACCTCCGATCCCTTTAACTTACTGCCTAAGCATACATAAGTTTTTAATATAGTTATATTGTCTAATTTGAA
ATCAATATCATCTTGAGCATGATATTTTACTATTAACATACATTTATTAACTGAGGAACAGTACTTGCACTTTCTTACATGCTTGCTCC
ATTCTATTATAATTTTACTCCAGTTGACTGTTACAGACAATTCGGCATCACAGACAAAAAGACTGATAGGTTCAGCAAACTTTATATG
TAAATAAGACCAATGAATGTTGTTGGTTGCATCTGTAGCAGGAATGGTCAAATTTTCACCATAATCAATGTTGATATGTCCATTGTAC
AGCCTTAAAAACTCAATAGGTAAACTATGATCATTGCAATCTTTCAGACTTCTGTAAATATATCTTATGTCAGGATGAAGTTCCACTAC
TGTACGCAATAATAAATTCCCTGCTCCTTCACCTATGAATGCTATACAATTGGGATCTTTAATTTTAAGATCTTTTAAAATATACTCTATA
CTAATTTTACAACCTGTAGAACTAAATACAAAATTGAATCTATTAATATGATGCCAAGGAAGCATGCAGTAAAGTGATGTGCTATTGTG
CACTAAAGATATTTGGTGGGAAGTAGTAGTGTAAAGTTGGTTGGATTTGGCTGTATTGCCTGAATGATCTATAATTCTATCAATCACA
ACCATAGGGAATAAATTATACAAATCTTGTTTGCTGTAATTGGTTCTAATCATTGCAGACGATTTAATAAGCTTCTTATTAGATAACAAT
GGT:::AATCTAATTGGTTTATATGTGTATTAACTAAATTACGA*GATATTAGTTTTTGACACTTTTTTTCTCG*

Fig2C: R2-10U L2 1250Bp
TAGGGTAACAAAGAAAGGGTATCAAACTTTTAATATTTGCATCAATAGACTCTTTATCAGCTTTCTTAGGCATGATGAAATTTTTGGT
TCTTGATAGTATCAATTTAGCATTTTGTACTACATTAAATACTGGGAATATATTCGCAGGACCTATTGTAAGGACTAAGTAAACCTCC
GATCCCTTTAACTTACTGCCTAAGCATACATAAGTTTTTAATATAGTTATATTGTCTAATTTGAAATCAATATCATCTTGAGCATGATAT
TTTACTATTAACATACATTTATTAACTGAGGAACAGTACTTGCACTTTCTTACATGCTTGCTCCATTCTATTATAATTTTACTCCAGTT
GACTGTTACGGACAATTCGGCATCACAGACAAAAAGACTGATAGGTTCAGCAAACTTTATATGTAAATAAGACCAATGAATGTTGT
TGGTTGCATCTGTAGCAGGAATGGTCAAATTTTCACCATAATCAATGTTGATATGTCCATTGTACAGCCTTAAAAACTCAATAGGTA
AACTATGATCATTGCAATCTTTCAGACTTCTGTAAATATATCTTATGTCAGGATGAAGTTCCACTACTGTACGCAATAATAAATTCCC
TGCTCCTTCACCTATGAATGCTATACAATTGGGATCTTTAATTTTAAGATCTTTTAAAATATACTCTATACTAATTTTAC:::ACACTAACA
CTGTATTCAATTATAGTTATTAAAAATTAAAAGTCGTACGATTTTTTAAATATTTTTTTTTTTTTTTAACTAATCCTAAAGTTATCATTTT
AATCTTGGAGGAATAAATTTAAACCCTAATCTAATTGGTTTATATGTGTATTAACTAAATTACGA*GATATTAGTTTTTGACACTTTTTT*
TCTCG

Fig2C: R2-10U L3 1200Bp
ATTAATTCCTTTTTTTGTTATAGGGTAACAAAGAAAGGGTATCAAACTTTTAATATTTGCATCAATAGACTCTTTATCAGCTTTCTTA
GGCATGATGAAATTTTTGGTTCTTGATAGTATCAATTTAGCATTTTGTACTACATTAAATACTGGGAATATATTCGCAGGACCTATT
GTAAGGACTAAGTAAACCTCCGATCCCTTTAACTTACTGCCTAAGCATACATAAGTTTTTAATATAGTTATATTGTCTAATTTGAAAT
CAATATCATCTTGAGCATGATATTTTACTATTAACATACATTTATTAACTGAGGAACAGTACTTGCACTTTCTTACATGCTTGCTCCA
TTCTATTATAATTTTACTCCAGTTGACTGTTACAGACAATTCGGCATCACAGACAAAAAGACTGATAGGTTCAGCAAACTTTATATG
TAAATAAGACCAATGAATGTTGTTGGTTGCATCTGTAGCAGGAATGGTCAAATTTTCACCATAATCAATGTTGATATGTCCATTGTA
CAGCCTTAAAAACTCAATAGGTAAACTATGATCATTGCAATCTTTCAGACTTCTGTAAATATATCTTATGTCAGGATGAAGTTCCAC
TACTGTACGCAATAATAAATTCCCTGCTCCTTCACCTATGAATGCTATACAATTGGGATCTTTAATTTTAAGATCTTTTAAAATATACT
CTATAC:::TACACTAACACTGTATTCAATTATAGTTATTAAAAATTAAAAATCGTACGATTTTTTAAATATTTTTTTTAACTAATCCTAAAGT
TATCATTTTAATCTTGGAGGAATAAATTTAAACCCTAATCTAATTGGTTTATATGTGTATTAACTAAATTACGA*GATATTAGTTTTTGAC*
ACTTTTTTTCTCG

Fig2C: R2-10U L3 700Bp
*CTTAGGTAAGGATATGTAGATTCTACC*TACACTGAGTTCTCTTTCTTTGCCTGTCAATGATACCACATGATTAGGGTTGTTGAG
ATAACTTTGATTAACTACACAGTTGTATAAATCACATTCATTGAATTTGTTATCTCTTAAATAATACTCTAATACTCTTCTTGATTTATC
ACTCTCGGAAAATTTTAATTTTTCATGTTCTATATAGTTTTGTATGTGTGATGGCATGTAATTTCTAGGGAAACTAGTCCATATCAAA
TTTTTAGGAGGTGATATAGCTTTATCATTTATAATCATTTCAAGATCCACTTTTCTAGGCAACCGAAACTCACGATAGAAACGTAGT
CCTGATAACACAATCAAATCTCTTTCTGTAAGTTCCAACAAAGAAGGATAAGTGTTTAGTTTATAGTAAGTTAACCATCTTAAGGGT
AAAACAATAGCATTTCTTAAAGTAGGCCATCTGTTGTAATTATTTACAAACCCTTTTATAATTCTATATATAAAGGCACCTCTTAAC:::A
AAATTAAAAATCGTACGATTTTTTAAATATTTTTTTTAACTAATCCTAAAGTTATCATTTTAATCTTGGAGGAATAAATTTAAACCCTA
ATCTAATTGGTTTATATGTGTATTAACTAAATTACGA*GATATTAGTTTTTGACACTTTTTTTCTCG*

Fig2E: WT L1 22Hr 900Bp
*CTTAGGTAAGGATATGTAGATTCTACC*ATATATAAATGGTTATAGTTTAGTTCTGTTGATCTGAAATTTAAAACATGATTGAACCATTT
TAAGATGTTCATATGCTTATGATTTATAAGTTTATTGCTGAAAACTTCATTACGTCCAGCTATAGAATATGATAGTATATCTCCACTAA
CAACACTCTTTAGTTTTGACAATGCAGTATTAATTCCTTTTTTTGTTATAGGGTAACAAAGAAAGGGTATCAAACTTTTAATATTTGC
ATCAATAGACTCTTTATCAGCTTTCTTAGGCATGATGAAATTTTTGGTTCTTGATAGTATCAATTTAGCATTTTGTACTACATTAAATA
CTGGGAATATATTCGCAGGACCTATTGTAAGGACTAAGTAAACCTCCGATCCCTTTAACTTACTGCCTAAGCATACATAAGTTTTTA
ATATAGTTATATTGTCTAATTTGAAATCAATATCATCTTGAGCATGATATTTTACTATTAACATACATTTATTAACTGAGGAACAGTACTT
GCACTTTCTTACATGCTTGCTCCATTCTATTATAATTTTACTCCAGTTGACTGTTACAGACAATTCGGCATCACAGACAAAAAGACT
GATAGGTTCAGCAAACTTTATATGTAAATAAGACCAATGAATGTTGTTGGTTGCATCTGTAGCAGGAATGGTCAAATTTTCACCATA
AACG:::ATTTTTTAAATAACTTTTAGTGAACTAATCCTAAAGTTATCATTTTAATCTTGGAGGAATAAATTTAAACCCTAATCTA
ATTGGTTTATATGTGTATTAACTAAATTACGA*GATATTAGTTTTTGACACTTTTTTTCTCG*

CACCTCCGATCCCTTTAACTTA -- Pair1-DI1 used for minigenome cbDVG RT-PCR
CTTAGGTAAGGATATGTAGATTCTACC -- RSVDI1 used for infection cbDVG RT-PCR
GATATTAGTTTTTGGACACTTTTTTTCTCG -- RevSmall used for minigenome and infection cbDVG RT-PCR
::: -- indicates break/rejoin junction position

Figure S2: Sequences of cbDVGs identified from indicated RT-PCR amplicons from viral infection and the RSV minigenome system. Italic font indicates the Small-Rev sequences used for cbDVG detection from viral infections and minigenome transfection experiments. Small-Rev was only used for PCR. Gray italic font indicates the Pair 1-DI-For and RSVDI1-For primer sequence used for cbDVG detection from RSV minigenome transfections and viral infections, respectively. ":::" indicates the location of the break and rejoin junction position. Pair 1-DI-For and RSVDI1 were used in both the reverse transcription and PCR steps, respectively.

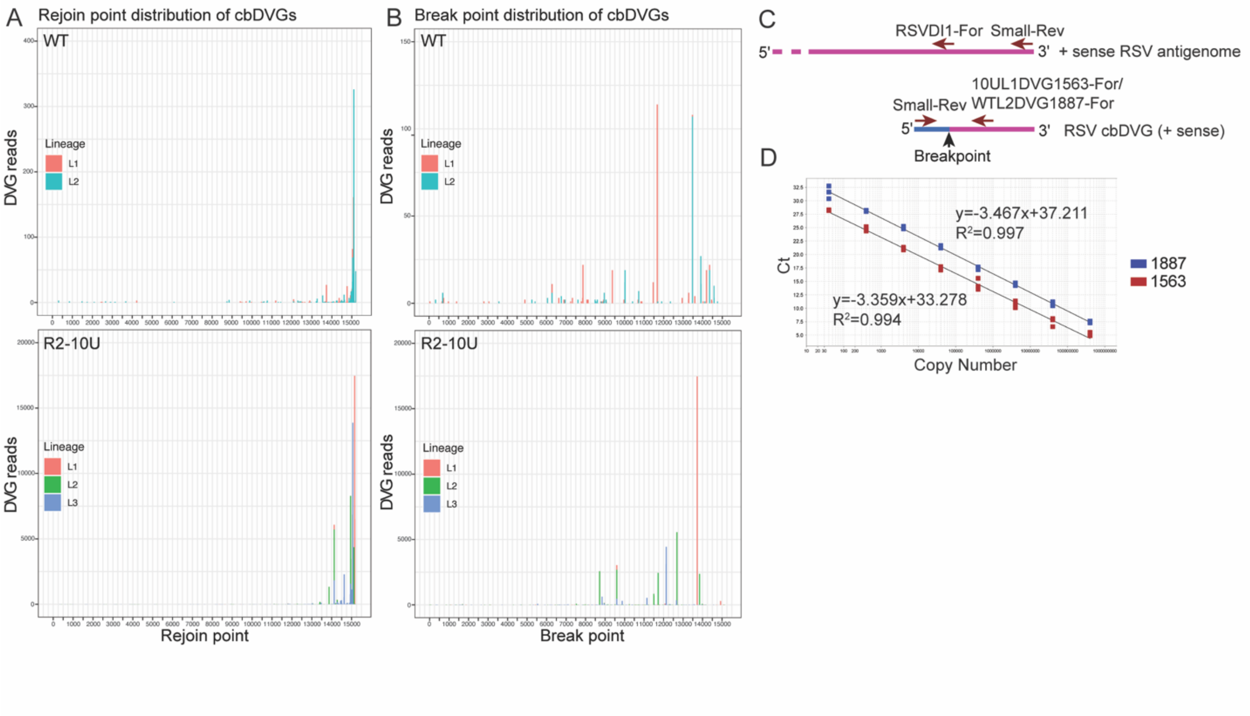

Figure S3: Genome-wide distribution of WT and R2-10U virus cbDVG break and rejoin points and qPCR strategy to quantify cbDVGs 1887 and 1563. (A) Histograms depicting the total number of cbDVG rejoin points at each genomic position of the WT (top) and R2-10U (bottom) HD P10 viruses spanning the entire genome. (B) Histograms depicting the total number of cbDVG break points at each genomic position of the WT (top) and R2-10U (bottom) HD P10 viruses spanning the entire genome. (C) cbDVG 1887 and 1563 RT-qPCR strategy. Briefly, RSVDI1 was used to reverse transcribe the (+) sense of 1887 and 1563, respectively. qPCR detection of 1887 and 1563 used Small-Rev and WTL2DVG1887-For and 10UL1DVG1563-For, respectively. (D) qPCR standard curves were determined by serial dilution of specific cbDVG encoding plasmids and was used to quantify the absolute copy number of (+) sense 1887 and 1563. Line equations are shown.

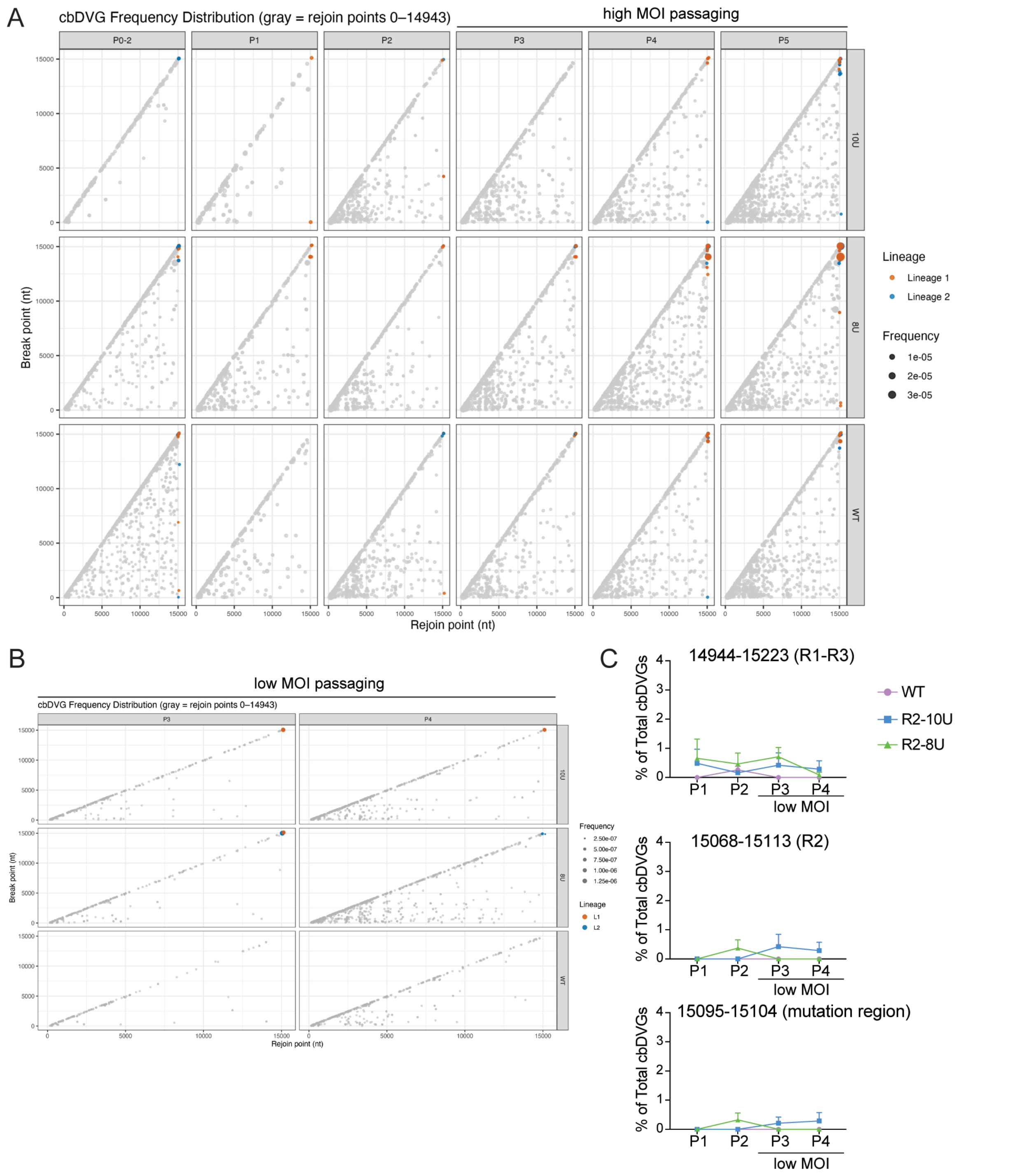

Figure S4: High and low MOI passaging genome-wide distributions of WT, R2-10U and R2-8U virus cbDVG break and rejoin point frequency. (A-B) cbDVG break and rejoin point distributions relative to viral genomic reads under high (A) or low (B) MOI passaging conditions. Each point represents a cbDVG species with the location indicating its break and rejoin point positions. The size of a given point indicates the number of cbDVG reads normalized to reads fully aligning to the viral reference genome for that given cbDVG. Colored

points indicate trailer cbDVGs (rejoin points within 14944-15223). (C) Under low MOI passaging conditions, the percentage of the trailer cbDVGs with rejoin points within R1-R3 (14944-15223, top), R2 (15068-15113, middle), or specifically within the R2-10U mutation sequence (15095-15104, bottom).

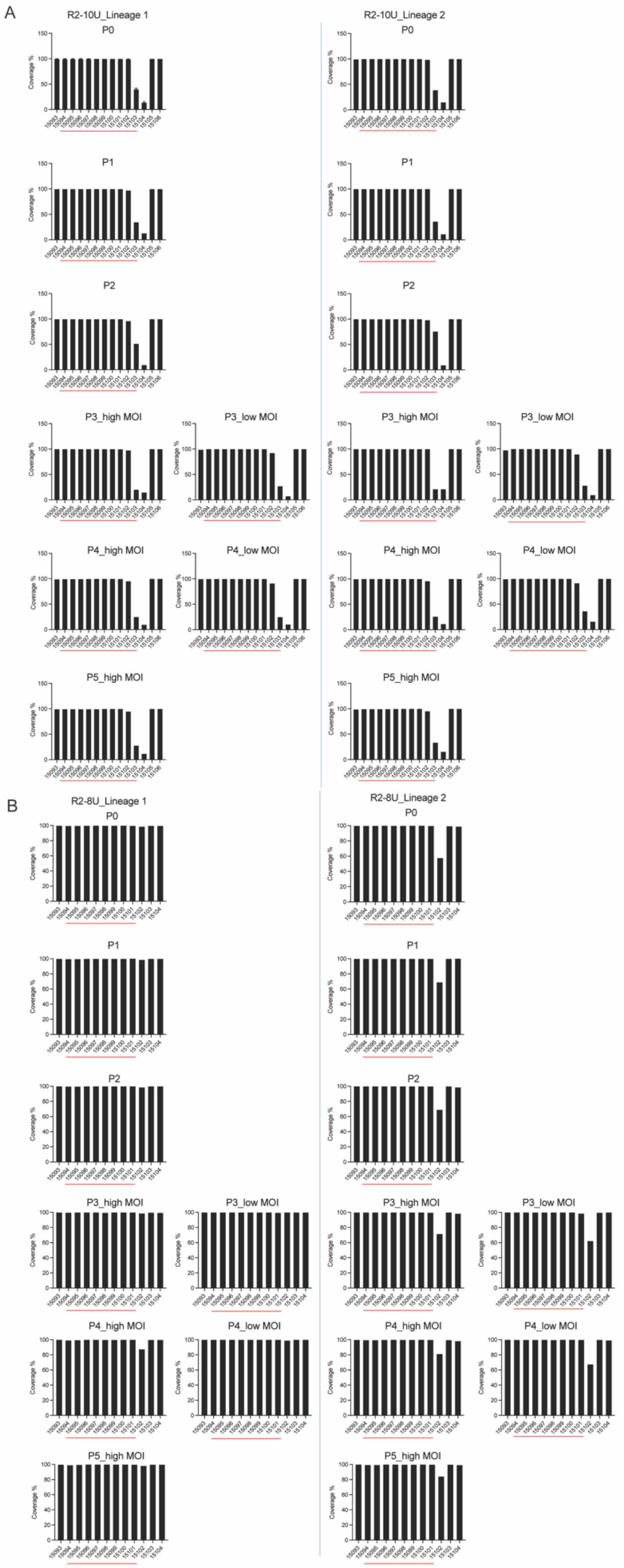
A
R2-10U_Lineage 1
R2-10U_Lineage 2
P0
P1
P2
P3_high MOI
P3_low MOI
P4_high MOI
P4_low MOI
P5_high MOI
Coverage %
B
R2-8U_Lineage 1
R2-8U_Lineage 2
P0
P1
P2
P3_high MOI
P3_low MOI
P4_high MOI
P4_low MOI
P5_high MOI
Coverage %

Figure S5: RNA-seq read coverage of the R2-10U and R2-8U mutation sequences. Sequencing read coverage of the R2-10U mutation sequence during R2-10U virus passaging (A) or the R2-8U mutation sequence during R2-8U virus passaging (B) normalized to viral reads aligning to the final 200 nucleotides of the viral genome. Red line indicates R2-10U and R2-8U mutation sequences, respectively.

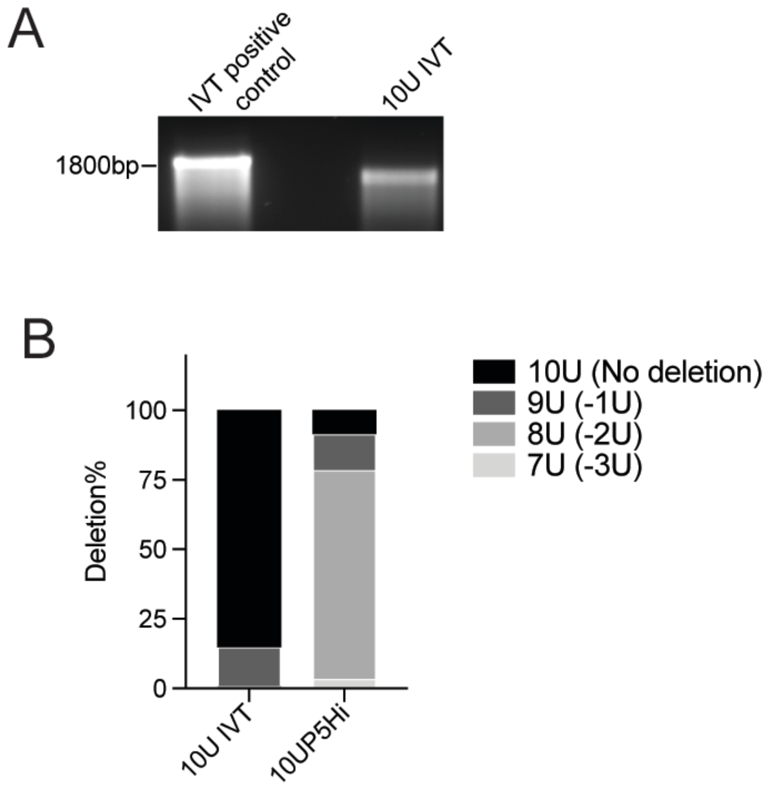

Figure S6: Assessment of sequencing error rates in the R2-10U region using IVT RNA. RNA containing the 10U mutation were generated by IVT and subjected to deep sequencing. (A) Agarose gel showing the IVT positive control RNA (~1.8 kb) and the IVT 10U RNA at the expected size (~1.2 kb). (B) IVT 10U RNA and 10UP5Hi RNA (internal control) were subjected to deep sequencing. Adapter-trimmed reads were aligned to the mutation region and the percentages of reads containing different numbers of Us were calculated from the aligned reads using the same R script as in Fig. 7A.

Table S1 Primer list

Primer name	Sequence (5’-3’)	Application
RSVDI1	CTTAGGTAAGGATATGTAGATTCTACC	cbDVG RT-(q)PCR
RevSmall	CGAGAAAAAAAGTGTCAAAAACTAATATC	cbDVG RT-(q)PCR, (-)genome qPCR, RSV trailer qPCR
Pair1-DI1	CACCTCCGATCCCTTTAACTTA	Minigenome cbDVG RT-PCR
NcoI-HhRbz-RevSmall	GAGACCATGGCTGATGAGTCCGTGAGGACGAAACGG AGTCTAGACTCCGTCACGAGAAAAAAAGTGTCAAAA ACTAATATCTCGTAATTTAG	cbDVG cloning
BsiWI-R	AAAAATCGTACGATTTTTAATTTTTAATAACTATAATT G	cbDVG cloning
BsiWI-2-R	AAAAATCGTACGATTTTTAATTTTTAATAACTATAATT GAATACAGTGTTAGTG	cbDVG cloning
15292-F	ATCGGAGGTTTACTTAGTCCT	cbDVG RT-qPCR
15760-F	GTTATACAACTTTCATAATGAA	cbDVG RT-qPCR
10UL1cbDVG156 3-F	TACAAATCTTGTTTGCTGTAATTGG	cbDVG RT-qPCR
WTL2cbDVG188 7-F	AGAAGTTATAATTAATGTAGAAGAGATTAG	cbDVG RT-qPCR
gRSV-R2-8U-F	TCAATTATAGTTATTAAAAATTAAAAATCGTACGATT TTTAAATATTTTTTTTAACTAATCCTAAAGTTATCAT TTTAATC	Site directed mutagenesis
gRSV-R2-8U-R	GATTAAAATGATAACTTTAGGATTAGTTAAAAAAAAT ATTTAAAAAATCGTACGATTTTTAATTTTTAATAACTA TAATTGA	Site directed mutagenesis
NcoI-ins-R1-SDM-F	CATCAGACGCGAAAAACCATGGTCTCCCTATAGTGAG TCGTATTAGCG	cbDVG cloning
NcoI-ins-R1-SDM-R	CGCTAATACGACTCACTATAGGGAGACCATGGTTTTT CGCGTCTGATG	cbDVG cloning
gRSV-R2-10U-F	CTCCAAGATTAAAATGATAACTTTAGGATTAGTTAAA AAAAAAATATTTAAAAAATCGTACGATTTTTAATTTT TAATAACTATAATTGAATACAGTGTTAGTGTATAGC	Site directed mutagenesis
gRSV-R2-10U-R	GCTATACACTAACACTGTATTCAATTATAGTTATTAA AAATTAAAAATCGTACGATTTTTAAATATTTTTTTTT TAACTAATCCTAAAGTTATCATTTTAATCTTGGAG	Site directed mutagenesis
human GAPDH-F	GCAAATTCCATGGCACCGT	qPCR
human GAPDH-R	TCGCCCCACTTGATTTTGG	qPCR
Human IFNB1-F	GTCAGAGTGGAAATCCTA AG	qPCR
Human IFNB1-R	ACAGCATCTGCTGGTTGAAG	qPCR
Human IL-29-F	CGCCTTGGAAGAGTCACTCA	qPCR
Human IL-29-R	GAAGCCTCAGGTCCCAATTC	qPCR
Human ISG56-F	GGATTCTGTACAATACACTAGAAACCA	qPCR

Human ISG56-R	CTTTTGGTTACTTTTCCCCTATCC	qPCR
Human MxA-F	GGCTGTTTACCAGACTCCGACA	qPCR
Human MxA-R	CACAAAGCCTGGCAGCTCTCTA	qPCR
Hamster-beta-actin-F	ACTGCCGCATCCTCTTCCT	qPCR
Hamster-beta-actin-R	TCGTTGCCAATGGTGATGAC	qPCR
RSV G-F	AACATACCTGACCCAGAATC	qPCR
RSV G-R	GGTCTTGACTGTTGTAGATTGCA	qPCR
RSV NS1-F	TGGCTAAGGCAGTGATACATACA	qPCR
RSV NS1-R	GCCATTAGGTTGAGAGCAGTG	qPCR
gRSVDI-F	CCTAAAGTTATCATTTTAATCTTGGAGGA	(-)genome qPCR, RSV trailer qPCR

Table S2 Mutations identified outside of the R2-10U sequence

Genomic position	WT viruses					
	L1_P5	L1_P10	L2_P5	L2_P10		
**2518**			U (20%)			
			A (80%)[a]			
**7469**			G (20%)			
			A (80%)			
**7554**	U (29%)					
	C (71%)					
**7734**			G (24%)			
			A (75%)			
**8868**			C (20%)			
			A (80%)			
**15534**		G (28%)		G (37%)		
		A (72%)		A (63%)		
**15535**		G (27%)		G (36%)		
		A (73%)		A (64%)		
**15536**		G (27%)		G (36%)		
		A (73%)		A (64%)		
**15611 (U to C)[b]**		C (80%)		C (83%)		
		U (20%)		U (17%)		
**15648 (U to C)**		C (81%)		C (85%)		
		U (19%)		U (15%)		
**15787**		C (23%)		C (22%)		
		U (77%)		U (78%)		
**15794**		C (40%)		C (41%)		
		U (60%)		U (59%)		
**15802**		C (41%)		C (42%)		
		U (59%)		U (58%)		
**15809**		G (37%)		G (35%)		
		A (63%)		A (65%)		
**15810**		G (37%)		G (35%)		
		A (63%)		A (65%)		
**15811**		G (37%)		G (35%)		
		A (63%)		A (65%)		
**15880 (A to G)**		G (55%)		G (53%)		
		A (45%)		A (47%)		
**15884**		G (26%)		G (25%)		
		A (74%)		A (75%)		

	R2-10U viruses					
	L1_P5	L1_P10	L2_P5	L2_P10	L3_P5	L3_P10
**7466**	G (25%)	G (20%)	G (38%)		G (38%)	
	U (73%)	U (77%)	U (60%)		U (61%)	
	A (2%)	A (3%)	A (2%)		A (1%)	
**12827**						C (74%)
						U (25%)
**15754**				U (78%)		
				G (22%)		

[a] Grey cells in the table contain the nucleotides in the reference genomes.
[b] Genomic positions highlighted in red indicate positions with a mutation greater than 50% (considered as major mutations).
